# Adoptive Cell Therapy—Harnessing Antigen-Specific T Cells to Target Solid Tumours

**DOI:** 10.3390/cancers12030683

**Published:** 2020-03-13

**Authors:** Elżbieta Chruściel, Zuzanna Urban-Wójciuk, Łukasz Arcimowicz, Małgorzata Kurkowiak, Jacek Kowalski, Mateusz Gliwiński, Tomasz Marjański, Witold Rzyman, Wojciech Biernat, Rafał Dziadziuszko, Carla Montesano, Roberta Bernardini, Natalia Marek-Trzonkowska

**Affiliations:** 1 International Centre for Cancer Vaccine Science (ICCVS), University of Gdańsk, 80-309 Gdańsk, Poland; elzbieta.chrusciel@ug.edu.pl (E.C.); zuzanna.urban-wojciuk@ug.edu.pl (Z.U.-W.); malgorzata.kurkowiak@ug.edu.pl (M.K.); jacek.kowalski@phdstud.ug.edu.pl (J.K.); 2 Department of Pathomorphology, Medical University of Gdańsk, 80-210 Gdańsk, Poland; biernat@gumed.edu.pl; 3 Department of Medical Immunology, Medical University of Gdańsk, 80-210 Gdańsk, Poland; mateusz.gliwinski@gumed.edu.pl; 4 Department of Thoracic Surgery, Medical University of Gdańsk, 80-210 Gdańsk, Poland; marjanski@gumed.edu.pl (T.M.); wrzyman@gumed.edu.pl (W.R.); 5 Department of Oncology and Radiology, Medical University of Gdańsk, 80-210 Gdańsk, Poland; rafal.dziadziuszko@gumed.edu.pl; 6 Department of Biology, University of Rome "Tor Vergata", 00133 Rome, Italy; montesan@uniroma2.it; 7 Department of Biology and Interdepartmental Center CIMETA, University of Rome "Tor Vergata", 00133 Rome, Italy; roberta.bernardini@uniroma2.it; 8 Laboratory of Immunoregulation and Cellular Therapies, Department of Family Medicine, Medical University of Gdańsk, 80-210 Gdańsk, Poland

**Keywords:** adoptive cell therapy of cancer, immunotherapy, TCR therapy, neoantigens, antigen- specific T cells, T cell-based therapy of solid tumours

## Abstract

In recent years, much research has been focused on the field of adoptive cell therapies (ACT) that use native or genetically modified T cells as therapeutic tools. Immunotherapy with T cells expressing chimeric antigen receptors (CARs) demonstrated great success in the treatment of haematologic malignancies, whereas adoptive transfer of autologous tumour infiltrating lymphocytes (TILs) proved to be highly effective in metastatic melanoma. These encouraging results initiated many studies where ACT was tested as a treatment for various solid tumours. In this review, we provide an overview of the challenges of T cell-based immunotherapies of solid tumours. We describe alternative approaches for choosing the most efficient T cells for cancer treatment in terms of their tumour-specificity and phenotype. Finally, we present strategies for improvement of anti-tumour potential of T cells, including combination therapies.

## 1. Introduction

Currently, the two main approaches dominate T cell-based therapies of cancer. These strategies harness: (1) native or genetically engineered T cells with antigen-specific T cell receptor (TCR); or (2) T cells genetically modified to express chimeric antigen receptor (CAR). Even though both approaches aim to induce antigen-specific reaction of T cells and may involve genetic modifications of the cells, significant differences in T cell responses can be observed for these strategies. They are a consequence of structural differences between CAR and TCR receptors.

The TCR is a T cell specific receptor that serves for antigen recognition by naturally occurring T cells, whereas CAR is an artificial chimeric receptor that combines both antigen-binding and T-cell activating functions. In addition, due to its unique structure and function, TCR can recognise only peptides bound to major histocompatibility complex (MHC) molecules, while CARs can potentially bind various types of antigens (not only peptides) and do not need MHC presentation. This can be an advantage in the case of MHC loss that is observed for many tumours [1]. Nevertheless, CARs bind surface antigens only, whereas TCRs can recognise all types of tumour-specific proteins processed into peptides and presented on MHC molecules, including intracellular proteins that remarkably increases the number of potential peptide targets.

## 2. TCR—Structure and Signalling

The role of TCR is to recognise antigenic peptides bound to MHC to elicit cell activation. During development in the thymus, adequate response to TCR stimulation is fundamental for positive and negative selection of T cells leading to development of T cell clones that can distinguish between self and non-self antigens [2]. TCR recognition of antigenic peptides is characterised by high level of degeneracy, meaning that one TCR can recognise multiple antigens and one antigen can be recognised by multiple TCRs with a broad range of affinities [3,4].

**Figure 1:**
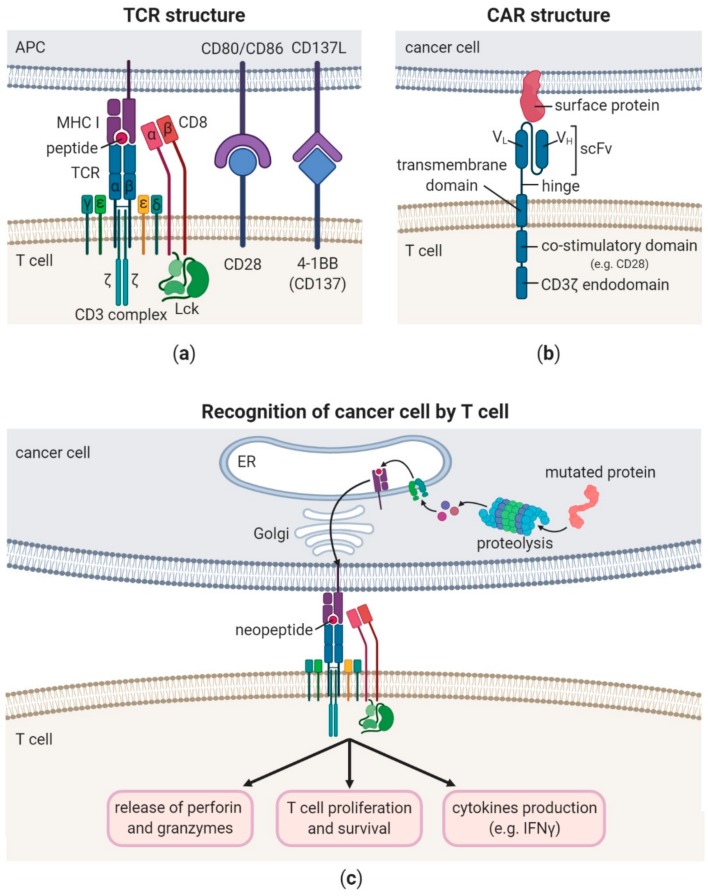
Comparison between T cell receptor (TCR) and chimeric antigen receptor (CAR) structure and signalling. (**a**) TCR interacts with CD3 complex (composed of two heterodimers CD3γ/CD3ε and CD3δ/CD3ε and a single homodimer CD3ζ/CD3ζ). TCR recognises and binds a peptide presented by a major histocompatibility complex (MHC) class I molecule. CD8 co-receptor stabilises this interaction and recruits lymphocyte-specific protein tyrosine kinase (Lck) to the TCR signalling complex, enhancing cytotoxic T cell activation. Activation of naïve T cell requires delivery of the second signal which is transduced by CD28 after binding co-stimulatory molecules CD80 or CD86 on the surface of antigen presenting cell (APC). Activated T cell upregulates expression of other co-stimulatory receptors (e.g., 4-1BB), that promote its effector functions, survival and proliferation. The figure depicts antigen recognition by CD8+ T cell in the context of MHC class I molecule. Antigen recognition by CD4+ T cell was not presented for the clarity. However, the processes of CD4+ and CD8+ T cell activation are analogous with the exception for the type of MHC that presents antigen (MHC class I and MHC class II for CD8+ and CD4+ T cells, respectively) and co-receptors that participate in cell activation (CD8 and CD4 for CD8+ and CD4+ T cells, respectively). (**b**) CAR consists of: a single-chain antibody variable fragment (scFv), composed of a variable light (V_L_) and variable heavy (V_H_) chain derived from a monoclonal antibody; an extracellular spacer region (termed hinge); a transmembrane domain; one or two co-stimulatory domains (e.g., CD28 and 4-1BB) for second-generation or third-generation CARs, respectively; and CD3ζ signalling domain. CAR binds a surface antigen via scFv (antibody recognition) in an MHC-independent manner. (**c**) The lower panel depicts antigen recognition in MHC restricted and TCR dependent manner by CD8+ T cell. This kind of antigen recognition can induce T cell responses against both surface and intracellular antigens of the cancer cell, as both are presented in the context of MHC molecules. Upon recognition of a tumour-specific antigen (neoantigen) or a tumour-associated antigen presented by a cancer cell, a cytotoxic CD8+ T cell activates and produces various proinflammatory cytokines, e.g., interferon γ (IFNγ), and releases perforin and granzymes, which lead to cancer cell death (the figure was created with BioRender software).

TCR is a heterodimer formed of either α and β (in over 90% of T cells) or γ and δ chains (in 1–10% of T cells) (Figure 1a) [5,6]. TCR lacks its own signalling domains, therefore it associates with CD3 complex that consists of two heterodimers CD3γ/CD3ε and CD3δ/CD3ε and a single homodimer CD3ζ/CD3ζ (also termed CD247). The CD3 complex contains a total of ten immunoreceptor tyrosine-based activation motifs (ITAMs), which are phosphorylated by lymphocyte-specific protein tyrosine kinase (Lck) leading to T cell activation [7]. To fully trigger T cell activation, the signal from the T cell complex (TCR + CD3 complex) has to be enhanced by simultaneous binding of the MHC molecules that present the antigenic peptide with a specific co-receptor CD4 or CD8. CD4 is present on the surface of T helper cells (Th) and binds to MHC class II molecules, while CD8 is expressed by cytotoxic T cells (Tc) and interacts with MHC class I receptors. CD4 and CD8 were reported to be involved in targeted delivery of Lck to the relevant TCR complex [8]. Signal from TCR complex is sufficient for activation of antigen experienced T cells (Figure 1c). However, cells that have not yet encountered specific antigen (naïve T cells) require the second signal, which is transduced by CD28 after binding co-stimulatory molecules CD80 or CD86 on the surface of antigen presenting cell (APC) (Figure 1a). This interaction leads to expression of anti-apoptotic and pro-survival genes via induction of phosphoinositide 3-kinase (PI3K) and RAS pathways [9]. However, there are also multiple activation induced co-stimulatory molecules known to promote survival, proliferation and effector function of already activated T cells. These molecules include inter alia: 4-1BB (CD137), OX-40 (CD134) and inducible T cell co-stimulator (ICOS/CD278) [10,11,12]. Following T cell activation and prolonged antigen stimulation, T cells are found to express increasing numbers of co-inhibitory molecules such as cytotoxic T-lymphocyte–associated antigen 4 (CTLA-4) and programmed cell death protein 1 (PD-1) to maintain peripheral tolerance and avoid immunopathology [13]. This neatly orchestrated mechanism is often dysregulated in cancer and can be a target for immunotherapy.

## 3. CAR—Structure and Signalling

T cells have recently been harnessed to target tumour associated antigens without direct engagement of TCR recognition and signalling. This was possible due to genetic modification and transduced expression of CARs. CAR T cells beside their original TCR express genetically engineered receptor (CAR) that gives them a new ability to target specific antigen. The receptor is chimeric because it is a synthetic single chain antigen receptor composed of four modules: (1) antigen-recognition domain; (2) an extracellular hinge region; (3) a transmembrane domain; and (4) intracellular T-cell signalling domain (Figure 1b). Antigen binding domain usually derives from the variable regions of a monoclonal antibody linked together as a single-chain variable fragment (scFv). There have also been several successful approaches to design non-antibody-based antigen-recognition domain in CARs. For full functionality, the antigen-recognition domain and extracellular hinge are bridged with intracellular signalling motifs via transmembrane domain anchored in plasma membrane. The earliest CARs, called the first-generation CARs, were equipped with only a single CD3ζ endodomain. However, as CAR T cell expansion and survival was limited with this approach, the second-generation CARs incorporated co-stimulatory domains (e.g., CD28, 4-1BB, CD27, CD40L, OX40, ICOS and CD244) proximal to the CD3ζ sequence. The third-generation CARs contain two or more co-stimulatory molecules. Thus far, studies did not show their superiority over the second-generation CARs [14,15]. Thus, current CAR T cell therapies intend to mimic native TCR activation pathways to overcome MHC restriction of TCRs. However, despite the constant increase in our understanding of T cell responses, all consequences of T cell genetic manipulation cannot be predicted. CAR T cell therapy has been associated with life-threatening adverse effects, such as cytokine release syndrome resulting from massive CAR T cell activation [16].

## 4. Challenges for CAR T Cell Therapies of Solid Tumours

Unlike TCR, CAR does not need MHC presentation of the target antigen. Therefore, CAR T cells can elicit antigen-specific response against the patient’s tumour regardless of tissue incompatibility [14].

Until now, two CARs have been approved by the Food and Drug Administration (FDA) for clinical use. They are both second-generation CARs targeting the B-cell lineage antigen CD19 [14,15,17]. Regulatory approval for other second-generation CARs targeting different B-cell markers, most notably B-cell maturation antigen (BCMA) [18,19], are anticipated in the coming years.

The clinical success of anti-CD19 CAR T cell in treatment of refractory pre-B cell acute lymphoblastic leukaemia and diffuse large B cell lymphoma [14,17] was possible because expression of CD19 is restricted to B cell lineage and is particularly high and frequent in these B cell malignancies [20,21]. However, while CAR T cell therapy has yielded remarkable efficacy for haematological malignancies, its effect against solid tumours is unsatisfactory. It is mostly due to the fact that solid tumours lack unique surface tumour-specific antigens (TSAs) and that tumour-associated antigens (TAAs) overexpressed on tumours are also shared with normal tissues [22]. Additional problems include T cell homing to the site of the disease, penetration of T cells into solid masses, overcoming immunosuppressive tumour microenvironment (TME) and limited CAR T cell persistence after infusion [23]. Multiple strategies for improving CAR T cell therapy efficacy against solid tumours have been extensively reviewed recently and are summarised in Table 1 [22,23,24,25,26].

As mentioned above, the main safety issue of CAR T cell immunotherapies is the release of excessive amounts of cytokines (such as TNFα or IFNγ) after their activation in vivo that leads to cytokine release syndrome (CRS), which can result in life-threatening multi-organ dysfunctions [55]. However, in the case of cytokine-expressing CAR T cells, the baseline cytokine level is low and it is elevated only upon antigen recognition on the tumour [23]. Additional safety concern is CAR T cell autonomous growth, which has been addressed by introducing “safety switches”. One of such approaches was based on activation of an inducible caspase 9 and rapid cell death after exposure to a synthetic drug [56].

The remaining issue of CAR T cell therapy is the loss of expression of targeted antigen by the tumour during the treatment [39,47]. It has been suggested that only patients with homogenous antigen expression on all cancer cells should be treated with this approach to avoid recurrence. An alternative could be targeting more than one antigen, which has been demonstrated for glioblastoma [57]. However, this approach still needs optimisation [58].

## 5. Antigens Used for T Cell-Based Therapy

Efficient T cell-based therapy targets specific antigens without side effects. A long list of tumour-associated antigens (TAAs) has been published recently [25]; however, most of them are also expressed at a low level on normal cells. Thus far, clinical trials with CAR T cells in solid tumours have struggled with severe toxicities as the targeted antigens were not specific to cancer cells [35,59,60,61]. A similar problem was observed in TAA-targeted TCR therapies. A prominent example was ACT with T cells recognising peptide derived from MAGE-A3, a member of melanoma-associated antigen (MAGE) family. These therapeutic T cells cross-reacted with MAGE-A12 expressed in the brain, resulting in severe adverse effects including patient’s death [62]. These results underline the importance of specific tumour targeting as even low expression of the target antigen on normal cells can lead to a severe toxicity.

The perfect targets for T cell-based therapies are tumour-specific antigens called also neoantigens, as they are highly immunogenic and not produced by normal tissues [63]. They can be either products of non-synonymous mutations in cancer genome or viral-specific proteins expressed by virally-induced tumours such as cervical and head and neck cancer associated with human papilloma virus (HPV) and Epstein-Barr virus (EBV) infection, respectively [64,65]. As there are relatively few types of cancer with known viral aetiology, most researchers focus on mutations as a source of neoantigens. The highest mutation frequencies occur in cancers caused by exposure to carcinogens such as ultraviolet (UV) radiation (melanoma) and tobacco smoke (lung cancers) [66]. Therefore, it is not surprising that most T cell-based clinical trials target melanoma [67]. Another interesting approach is the use of personalised neoantigen vaccines, also already tested in phase 1 clinical trial for melanoma [68]. Studies that attempted to screen cancer genome defined very few neoantigen-generating mutations, which makes them difficult targets of personalised therapy [69,70]. Nevertheless, deep sequencing of the surgically retrieved tumours and identification of neoantigens is not a routine clinical practice therefore the real scale of neoantigen generation in human tumours is not yet fully determined. In addition, previous and current studies of tumour genome screen big cohorts of patients and try to find at least a single neoantigen-generating mutation that would be universal for the majority of the studied cancer cases. High diversity of cancers (even within the same tumour type), high tumour mutational burden (TMB) together with unpredictability of these mutations abrogates discovery of this universal neoantigen, as it may not exist. Thus far, it appears that, in the case of therapies that target neoantigen, a personalised approach can be the only solution to define this neoantigen and design anti-cancer treatment.

To our knowledge, a splice variant of a mutated variant III of epidermal growth factor receptor (EGFRvIII) is the only example of a mutated surface protein targeted by CAR T cell therapy [46,47]. Therefore, targeting neoantigens is mainly in the scope of TCR therapy. Recently, alternative sources of neoantigens, such as RNA transcription and splicing errors leading to frameshift mutations have been also identified [71].

## 6. Advantages of TCR vs. CAR T Cell Therapy

Conventional CAR T cells recognise antigens expressed on the cell surface while TCRs have the advantage of targeting any peptide including those derived from intracellular protein degradation. Although recently a TCR mimetic CAR recognising NY-ESO-1/HLA (New York esophageal squamous cell carcinoma 1/human leukocyte antigen) complex has been developed, recognition of MHC-bound peptides is a characteristic of TCR therapy [72]. Therefore, the variety of antigens that can be recognised by TCR is much higher than by CAR.

Although it is difficult to directly compare CAR and TCR sensitivity due to differences in their structures, a recently developed single-chain TCR allowed that [73]. Full length TCR had greater sensitivity than CAR even when CARs were expressed at higher densities [7,67]. At the same time, TCRs were found to mediate release of lower cytokine levels [74]. Thus, the risk of cytokine release syndrome is potentially lower with TCR therapy, compared to CAR T cell approaches.

Overall, thus far, it appears that TCR therapy is more suitable for solid tumour treatment, than CAR T cells, especially due to the very limited number of tumour-specific surface antigens.

## 7. Strategies for Selection of T Cells for Cancer Therapy

### 7.1. Selected Ex Vivo Expanded Tumour-Reactive TILs vs. Unselected TILs After Shortened Expansion

The concept of utilising autologous tumour infiltrating lymphocytes (TILs) as an anticancer immunotherapy has been developed by Rosenberg and his group [75,76]. Patients with metastatic melanoma were subjected to lymphodepleting chemotherapy and then treated with autologous, ex vivo expanded TILs that showed specific reactivity against the tumour [77,78,79]. Long-term follow-up of the patients enrolled in clinical trials showed the overall response rate and complete response rate of 49–72% (depending on the additional treatment regimen) and 22%, respectively. Ninety-five per cent of the complete responders kept this status over three years of the follow-up [80]. An important limitation of this approach is that autologous tumour cells are required for identification of tumour-reactive TILs, which are not always available in therapeutically relevant numbers. Therefore, the Rosenberg’s group modified the protocol eliminating an assay for tumour recognition that was found to prolong the cell production significantly [81]. Additionally, they focused on enrichment for tumour reactive CD8+ T cells, elimination of non-specific CD4+ T cells [82,83], and depletion of regulatory T cells (Tregs) that are known of their potent immunosuppressive activity [84,85,86]. With this protocol modification, they demonstrated that selection of tumour-reactive cells is not required for effective ACT with TILs in melanoma patients, provided that TILs are expanded no longer than 2–3 weeks [81,82,83]. The short-term expansion ex vivo seems to be in general crucial for quality of all T cell subsets when adoptive transfer is considered, as confirmed also by our studies on Tregs [87]. A similar approach for TIL culture protocol design was tested by Itzhaki and co-authors. In this study, unselected TILs generated in short-term cultures (so called “young” TILs) induced clinical response in 48% of melanoma patients [88]. These both studies indicated that polyclonal TILs can be as efficient as selected tumour-reactive TILs in therapy of melanoma when expanded for a short time. Several medical centres worldwide performed clinical trials reproducing and improving an approach based on either selected or young TILs and demonstrated objective responses in approximately 40% of metastatic melanoma patients [89,90,91,92,93].

High therapeutic effectiveness of ACT with TILs in metastatic melanoma prompted investigators to test whether a similar approach would result in regression of other solid tumours. In a pilot study, ACT with TILs of patients with metastatic ovarian cancer resulted in a slight decrease of tumour size (maximally 23%) in five of six patients, but the effect was transient [94]. One of the reasons of a relatively modest therapeutic effect might be a low frequency (3%) of tumour-reactive T cells in ex vivo expanded TIL cultures [95]. Functional characterisation of expanded TILs from renal cell carcinoma and gastrointestinal cancer (GI) also revealed lower frequency of tumour-specific T cells compared to melanoma [96,97]. This can be an effect of relatively low mutational burden of aforementioned tumours and in consequence their low immunogenicity. Applying a method for selection and enrichment of tumour-recognising T cells directly from a tumour sample could improve efficiency of ACT in case of low mutational load epithelial cancers. It would prevent potential overgrowth of nonspecific T cells during ex vivo expansion, thus resulting in higher frequency of tumour-responsive T cells in the final product. This approach might also improve a response rate in melanoma patients. Several molecules have been used as biomarkers to specifically identify and select tumour-reactive T lymphocytes both in case of melanoma and epithelial cancers.

### 7.2. Enrichment of T Cells Expressing Markers of Activation

A co-stimulatory surface receptor CD137 (4-1BB) was proposed to serve as a biomarker of antigen-specific activation of T cells that enables identification of tumour specific T cells without knowledge of the target antigen [98]. Powell’s group showed that isolated CD137^+^ T cells isolated were highly tumour-reactive. Moreover, in preclinical studies they demonstrated that CD137-enriched TILs were more potent in tumour growth inhibition compared to the total TIL repertoire or CD137^-^ population [99]. Subsequently, a simple and rapid method for selection of CD137-expressing TILs was developed and validated for clinical purposes. CD137-selected TILs demonstrated high in vitro anti-tumour activity and were enriched in T cells recognising neoantigens and shared tumour antigens [100].

Interestingly, an inhibitory programmed cell death-1 (PD-1) surface receptor was shown to be a promising alternative for CD137. Due to chronic exposure to tumour antigens TILs start to express PD-1, show signs of immune exhaustion and have impaired anti-tumour activity. However, sorted PD-1+ TILs were found to downregulate PD-1 expression and regained their anti-tumour function upon in vitro culturing with IL-2 [101]. This suggests that PD-1 expression can be modulated by the environment and thus T cells may escape PD-1/PD-L1 suppression. Importantly, tumour-specific TILs were found in both PD-1+/4-1BB^+^ and PD-1+/4-1BB^-^ sorted subsets, indicating that PD-1 allows for identification of a larger repertoire of tumour-reactive TILs than 4-1BB [102]. Together, these results suggest that PD-1 might be a useful biomarker for tumour-reactive TILs enrichment. This concept was supported by the studies on murine model of melanoma, indicating that treatment with sorted and expanded PD-1+ TILs improved ACT efficacy compared to unsorted TILs [103].

Nevertheless, the efficiency of both 4-1BB and PD-1-based enrichment approaches for treatment of metastatic melanoma patients has to be evaluated in clinical trials.

### 7.3. Selection of TAA-Specific T cells

Apart from studies of polyclonal tumour-recognising TILs, the effectiveness of selected tumour antigen-specific TIL clones in melanoma treatment is also being investigated.

Adoptive treatment with identified and expanded T cell clones reactive to melanoma-associated antigen-recognised by T cells (MART-1) or glycoprotein 100 (gp100) resulted in positive, however, rather modest clinical responses [104]. Importantly, in three of five patients analysed, selective loss of targeted antigen occurred. Similar effects were observed by Mackensen and collaborators, who also used MART-1 specific T cells for therapy [105]. Their treatment strategy resulted in three responses (including one complete regression) in 11 patients. However, a selective loss of MART-1 expression was also observed in non-responding patients. These data provided rationale for targeting multiple tumour antigens simultaneously for melanoma treatment.

### 7.4. Selection of Neoantigen-Specific T Cells from the Tumour

The most recent strategies for selection of T lymphocytes for ACT are based on neoantigen specificity. Neoantigens are presented exclusively by cancer cells; therefore, the risk of on-target/off-tumour recognition is much lower than in the case of tumour associated antigens expressed also by normal cells. Contrary to TAA-specific T cells, T cells binding neoantigens with high affinity are not eliminated by the mechanisms of central tolerance. It was revealed that long-term complete tumour regression correlated with the presence of neoantigen-specific T cells, rather than TAA-reactive ones, within TILs adoptively transferred to the patients [106,107,108]. Interestingly, neoantigen-specific T cells were observed in the peripheral blood of patients even five years following ACT [107]. These results suggest that neoantigen-specific T cells play a crucial role in tumour regression.

Robbins and co-authors developed a strategy for identification of mutated antigens. It includes three stages: (1) whole exome sequencing (WES) of DNA isolated from matched tumour and normal (e.g., blood) sample; (2) in silico peptide prediction; and (3) experimental analysis of immunogenicity of predicted neoantigens [108]. This relatively rapid and simple approach was an attractive alternative to laborious cDNA library screening.

Currently, peptide prediction algorithms use either solely the data generated by mass spectrometry (MS) analysis of eluted MHC-binding peptides or both in vitro MHC-peptide binding affinity and MS peptidome data [109,110,111].

Identified candidate neopeptides have to be subsequently analysed for their immunogenicity. There are several methods evaluating which of the predicted neopeptides are recognised by the patient T cells. The first group of these methods is based on measurement of T cell activation in co-culture with MHC-matched cells expressing selected neoantigens. Usually, peptide-pulsed autologous dendritic cells or MHC-matched T2 cells are used. Alternatives for pulsed synthetic peptides are tandem minigenes (TMGs) [112]. TMGs are genetic constructs that encode several minigenes, each containing an identified somatic mutation flanked on both sides by the 12 amino acid residues specific for a wild-type protein. In vitro transcribed TMG RNAs are transfected into autologous APCs, allowing for the processing and presentation of mutated epitopes. Typical assays evaluating activity of T cells co-cultured with autologous APCs are: (1) measurement of IFNγ release by enzyme-linked immunosorbent assay (ELISA) or enzyme-linked immunospot assay (ELISPOT); and (2) measurement of 4-1BB expression by flow cytometry. The second type of methods used for identification of neoantigen-specific T cells utilises peptide-MHC (pMHC) multimers. Interaction of a T cell with purified peptide-MHC complex is very transient and its detection had not been feasible until 1996, when it was shown that tetramerization of peptide-MHC class I molecules provided sufficient stability to T cell receptor (TCR)-pMHC interaction [113]. pMHC multimers were labelled with fluorophores that enabled detection of T cells bound to pMHC multimers by flow cytometry. This technology allowed detection of neoantigen-specific T cells independently of their functional activity and presence of APCs. Since then, several important advances have been made, which enabled high-throughput identification of neopeptide-specific T cells, including: (1) UV mediated peptide exchange technology [114]; (2) combinatorial pMHC multimer staining [115]; (3) pMHC multimers labelled for mass cytometry analysis [116]; and (4) DNA barcode-labelled pMHC multimers [117,118].

Neoantigen-specific T cells can be identified in patients not only with melanoma, but also with epithelial cancers, such as lung cancer [119,120], gastrointestinal cancer [121,122,123], ovarian cancer [124,125,126], breast cancer [127] and pancreatic cancer [128]. Case reports have demonstrated that treatment of the gastrointestinal and breast cancer patients with a T cell population highly enriched in neoepitope-specific T cells caused tumour regression [121,123,127]. These results provide evidence that neoantigen-specific T cell-based therapy can result in tumour regression even in the case of low mutation load, when tumour-reactive T cells are scarce [129].

### 7.5. Selection of Neoantigen-Specific T Cells from Peripheral Blood

Rosenberg’s group demonstrated that neoantigen-specific T cells could be identified and isolated not only from the tumour, but also from the peripheral blood of melanoma patients [130,131]. Remarkably, neoantigen-specific T cells can be identified also in the peripheral blood of the patients with tumours harbouring relatively low number of mutations such as epithelial cancers [124,132,133]. Detection of rare neoantigen-specific T cells in the peripheral blood can be enhanced by using combinatorial pMHC multimer staining [130]. Additionally, several methods were developed to avoid a decline in their frequency upon expansion, due to overgrowth of non-reactive T cells. The first one, T cell library approach, was based on generation of more than a thousand parallel, small-scale T cell cultures, instead of expanding a bulk T cell culture from patient’s peripheral blood [132,134]. In the second approach, peripheral blood T cells expressing PD-1 and 4-1BB were sorted, followed by limiting dilution and microwell culture [135] or bulk expansion [133]. Alternatively, to stimulate growth of T cells recognising mutated peptides, peripheral blood T cells were expanded in the presence of pools of predicted neopeptides or dendritic cells loaded with tandem minigenes [124,136]. Due to these expansion strategies neoantigen-specific T cells can be obtained in frequencies sufficient for detection by standard screening methods: (1) co-culture with APCs presenting neopeptides, followed by interferon (IFN)-γ ELISPOT or flow cytometry analysis of 4-1BB expression; and (2) peptide-MHC multimer staining.

Strategies for identification of neoantigen-specific T cells in peripheral blood, combined with methods for generating TCR-engineered T cells, opened a possibility to develop a non-invasive approach for generation of neoantigen-targeted T cell-based therapies.

### 7.6. Selection of T Cells Recognising Neoantigens with Driver Mutations

Neoantigen-specific T cells are an attractive tool for cancer therapy, however several limitations exist. Cancer cells tend to accumulate novel mutations in the course of tumour growth, resulting in a mosaic pattern of the tumour. Hence, targeting only a single neopeptide may be an inefficient treatment strategy and lead to elimination of only a subset of tumour cells. To circumvent this problem, a heterogenous population of several distinct neoantigen-specific T cell clones could be infused. Alternatively, T cells targeting neoantigens derived from driver mutations could be used, as these mutant genes are likely to be expressed by all tumour cells. Recently, T cells recognising neopeptides derived from KRAS G12D in colorectal cancer [123,136], BRAF V600E in melanoma [137], histone 3 variant 3 H3.3K27M in glioma [138] and p53 hotspot mutations in various types of epithelial cancer [125,139,140] were identified, rising a possibility that other immunogenic neopeptides originated from hotspot driver mutations exist and can be used as targets for T cell-based therapies.

### 7.7. Genetic Engineering of T Cells to Express Specific TCR

Neoantigen-specific T cells identified and isolated from tumour or peripheral blood can be potentially expanded to therapeutically relevant numbers. However, it is not always possible, as the initial cell number can be very low (number of infiltrating leukocytes varies between the tumours and is limited by size of the resected tissue). In addition, proliferative potential TILs can be severely affected by the tumour and its environment, as we have observed for tumour-derived T cells in non-small cell lung cancer (NSCLC; unpublished data). Therefore, an alternative approach has been developed. The TCR sequence of sorted neoantigen-specific T cell clone can be determined. Subsequently, T cells isolated from patient’s peripheral blood are transfected with the sequence of this identified TCR with retroviral construct and expanded in vitro. This strategy was initially developed for generation of T cells specific for melanoma-associated antigens and resulted in complete tumour regression in two out of 15 patients [141]. Although the response rate was lower compared to the treatment with bulk TILs, this work paved the way for development of new strategies for ACT with TCR-modified T cells.

The main problem of TCR gene transfer with viral vectors into T cells is that this approach results in insertion of an additional copy of TCR gene instead of replacing the native one. The endogenous TCRs compete with transgenic ones for CD3-binding sites and thus for surface expression. Moreover, they can form mixed heterodimers, with altered, unpredictable and potentially autoreactive antigen specificity. To prevent mispairing, several approaches were developed, including the introduction of an additional disulphide bond between introduced TCR α and β constant domains [142,143,144] and linking TCR α and β chains with a self-cleaving 2A peptide [145]. More recent approaches utilised CRISPR-Cas9 technology to replace endogenous TCR with exogenous one via: (1) knockout of endogenous TCR gene simultaneously with transduction with a desired TCR [146] or (2) knock-in of exogenous TCR into a TCR-α constant (TRAC) locus [147,148]. Safety and efficacy of ACT with TCR-engineered T cells is now explored intensively in clinical trials, and importantly, most of them focus on solid tumours (over 80%) [67,149].

More than 40 clinical therapies based on TCR-engineered T cells target cancer testis antigens, whereas only three of them aim to induce immune response against neoantigens. Neoantigens are typically unique for a particular tumour derived from a particular patient, thus development of such a personalised T cell therapy is logistically complex and costly. These issues presumably limit the interest of biotechnology and pharmaceutical companies in ACT with neoantigen-specific T cells. However, the latest discoveries of T cells recognising neoantigens derived from hotspot mutations, that are frequent in many types of cancers (e.g., hotspot mutation within p53 gene), raised the possibility of development of cancer specific T cell products that will be available off-the-shelf [125,138,140].

## 8. Role of CD4^+^ Helper T Cells in an Anti-Tumour Response

Most studies concerning ACT focus on an anti-tumour activity of CD8+ T cells. However, accumulating evidence reveals that CD4+ T helper (Th) cells have an important role in anti-tumour response [150,151]. The major role of CD4+ Th cells is to support development, proliferation and effector function of CD8+ T cells. They exert this function both indirectly, by stimulation of APCs and thus promoting CD8+ T cells priming, and directly, by producing cytokines, mainly IFNγ and IL-2. Neoantigen-specific CD4+ T cells were found to infiltrate epithelial tumours [135,137,152]. It was reported that apart from acting on CD8+ T cells within lymph nodes, they elicit effective anti-cancer response at the tumour site [153]. CD4+ Th cells can recognise tumour cells via binding to MHC class II molecules (MHC II) complexed with neoantigens [154]. However, in most epithelial tumours, expression of MHC II is very low or not observed at all [155]. Abelin and co-authors suggested that, within tumour microenvironment (TME), CD4^+^ Th cells are mainly activated by resident APCs, which present neoantigens, derived from endocytosed tumour proteins, in the context of MHC II. It suggests that loss of MHC II expression by cancer cells can be less frequent mechanism of immune escape, than loss of MHC class I molecule (MHC I). Therefore, Th cells should not be ignored as a potential tool for cancer immunotherapy.

Animal studies showed that combined transfer of tumour-specific CD8+ Tc and CD4+ Th cells significantly enhanced anti-tumour response, suggesting that both T cell subsets act synergistically and improve effectiveness of ACT in cancer patients [156,157]. Only two clinical studies analysed the effects of ACT with neoantigen-specific CD4+ Th cells, but the results were encouraging. Adoptive transfer of NY-ESO-1-recognising CD4+ T-cell clone led to complete remission in a melanoma patient [158]. Tumour regression of cholangiocarcinoma was demonstrated following treatment with TILs highly enriched in neoantigen-specific CD4+ T cells [121]. Development of neoantigen-specific CD4+ T cells-based therapies is hampered by the complexity of MHC II processing and presentation, making MHC II-presented antigens difficult to identify. However, very recently, a new MHC II peptide prediction algorithm was developed [155]. Hopefully, these more accurate prediction tools will enable to establish therapies based not only on CD8+ Tc cells but also on CD4+ Th cells.

## 9. MR1-Restricted T Cells—A Novel Population of T Cells with Pan-Cancer Therapeutic Potential

Very recently Crowther and colleagues published results that opened an exciting prospect of potential use of MR1-restricted T cells in immune therapy not limited by MHC polymorphism [159].

MHC class I-related (MR1) molecule is a non-polymorphic MHC class I-like protein expressed at low levels on the surface of a wide variety of cells. Initially, it was demonstrated that MR1 presents microbial metabolites to T cells that harbour MR1 binding TCRs, allowing for recognition of infected cells [160,161].

In 2017, Lepore and co-authors identified in human blood a novel population of MR1-restricted T cells that recognise cancer cells via MR1 presentation [162]. These T cells demonstrated T helper-like capacities, as they were able to produce diverse cytokines, including IFNγ and IL-2, and induce maturation of dendritic cells.

Subsequently, Crowther and co-authors identified TCR that recognises nonbacterial antigen presented by MR1 expressed on the surface of cancer cells [159]. They demonstrated that the T cells harbouring this TCR were able to kill a wide variety of cancer cells in an MHC-independent manner. Importantly, these T cells did not recognise healthy cells from various analysed tissues. To test therapeutic potential of T cells targeting MR1-restricted antigens presented by cancer, the researchers adoptively transferred these cells into human leukaemia xenogaft mouse model. They observed regression of leukaemia and prolonged survival of mice. Moreover, Crowther et al. demonstrated in in vitro experiments that T cells of melanoma patients transduced with identified TCR could effectively kill not only the patient’s own cancer cells, but also non-autologous melanoma cells [159]. These data suggest that the MR1-restricted TCR, recognises diverse types of tumours irrespective of MHC and thus may open the opportunity for a pan-cancer T cell-based therap. Nevertheless, better understanding of the mechanism of action of MR1-restricted T cells and identification of cancer-associated ligands presented by MR1 is crucial for reaching this goal.

## 10. Choosing the Most Effective T Cell Differentiation State for ACT

Although tumour-antigen specificity is crucial for successful ACT, other features of T cells may also affect efficacy of this treatment. An important factor determining durability of therapeutic effect of ACT is the persistence of infused T cells.

The differentiation state is an important determinant for the longevity and therapeutic effect of infused T cells. Mature T cells that leave the thymus and had no contact with their specific antigen yet are called naive T cells (Tn). During antigen priming Tn expand and differentiate into effector cells (Teff) which reach sites where the recognised antigen can be found and activate machinery for the elimination of the antigen-bearing target. During prolonged antigen stimulation the majority of pathogen-specific Teff die via apoptosis. However, according to the classical theory of memory T cell generation about 5–10% of Teff differentiate into memory T cells (Tm) that survive. Among Tm, two distinct subsets can be recognised: (1) central memory (Tcm) T cells; and (2) effector memory (Tem) T cells. It has been widely accepted that upon antigen priming Tn differentiate progressively into Tcm and then Tem. After antigen re-stimulation, both Tcm and Tem proliferate and differentiate into short lived Teff that—again—actively mediate antigen specific responses [163,164,165]. Thus, Tn, Teff, Tcm and Tem differ in phenotype, homing and function, and they present a distinct therapeutic potential. It has been observed that less-differentiated T cells (Tn and Tcm) exhibit higher therapeutic potential than Tem and Teff. In addition, Tn and Tcm infused to the patients survive longer than Tem and Teff [164,166,167,168]. This provided rationale for establishment of a protocol for so called young TILs generation, via short-term culture ex vivo [81]. TILs obtained with this protocol are less differentiated and exhibit higher proliferative potential. Nevertheless, the majority of TILs isolated for expansion are already terminally differentiated and functionally exhausted, as a result of persistent antigen stimulation within the tumour site. Therefore, an alternative strategy was proposed, based on isolation of Tn or Tcm from patient’s peripheral blood mononuclear cells (PBMCs) and their genetic modification with desired TCR gene, followed by ex vivo expansion [169,170]. Klebanoff and co-authors provided rationale for this approach, showing that ex vivo expansion of Tn population gave rise to more therapeutically potent cells as compared to cultures where mixture of naive and memory subsets were used for expansion [171]. Recently, the dogma of T cell differentiation after antigen recognition and memory cell generation has been revised. It has been postulated that CD45RA+CD62L+ T cells, initially identified as Tn subset, are an amalgamation of naive and memory stem T cells (Tscm). In humans, Tscm were identified as CD45RA+CD45RO^-^ cells expressing high levels of lymph node-homing receptors CD62L and CCR7, as well as IL-7 receptor α-chain (IL-7Rα/CD127) and the co-stimulatory molecules CD27 and CD28. However, unlike Tn cells, Tscm express memory markers such as CD95, CD122, CXCR3, IL-2Rβ, CD58 and CD11a. Tscm have high proliferative capacity and are characterised by both self-renewal and ‘multipotency’ in that they can further differentiate into other subsets including Teff, Tcm and Tem cells. In light of these observations, it has been suggested that Tscm comprise an attractive tool for ACT [171,172]. A mouse study demonstrated that these cells are more effective in an anti-tumour response than Tcm, as their infusion resulted in a durable tumour regression [173]. The major limitation of their use is the fact that naturally occurring Tscm are rare (2–3% of circulating T lymphocytes) [174]. However, a method for in vitro differentiation, expansion and gene modification of Tscm from naive precursors isolated from PBMCs has already been published [175].

## 11. *Ex Vivo* T Cells Expansion and Artificial Antigen-Presenting Systems

Expansion of T cells for ACT is a challenge, as high cell numbers need to be generated while avoiding terminal differentiation.

Optimal T cell activation requires TCR stimulation and co-stimulation via CD28 molecule. In physiological conditions, these signals are provided by professional APCs. In vitro anti-CD3 antibodies are used to mimic antigen-mediated TCR signalling. These antibodies provide effective stimulus, when bound to Fc receptor-bearing APCs called also feeder cells, such as monocytes/macrophages and B cells. In addition, the feeder cells in this system constitutively express co-stimulatory molecules that target CD28, thus providing simultaneously two signals required for T cell activation and proliferation.

Therefore, the first protocols established for robust expansion of tumour-reactive TILs, called rapid expansion protocols (REP), included anti-CD3 (OKT3) antibodies and irradiated allogeneic PBMCs isolated from healthy donors (as feeder cells). In this system PBMCs were mixed with TILs in 200:1 ratio in the presence of high concentrations of IL-2 to obtain more than 1000-fold expansion. This protocol was based on stimulation of multiple potentially tumour reactive T cells in tumour independent manner and without determination of the target tumour antigens. In consequence multiple T cell clones proliferated, resulting in clinically relevant numbers of T cells that could be infused to the patients [78,80].

However, due to safety reasons and need of standardisation, alternative approaches for T cell stimulation were developed and irradiated PBMCs were substituted with artificial cell-based and non-cell-based antigen presenting systems [176,177,178].

In 2002, K562 cell line-based artificial APCs (aAPCs) were developed for ex vivo expansion of T lymphocytes Maus [177]. This erythromyeloid cell line does not express MHC molecules, which prevents allogenic T-cell responses. Thus, K562 cells were genetically engineered to stably express CD32/FCGR2A (Fc receptor) that allows for their coating with anti-CD3 and anti-CD28 antibodies. The studies with irradiated K562-derived aAPCs demonstrated that these cells promoted rapid and long-term expansion of T cells. Subsequently, K562-based aAPCs were additionally engineered to express a variety of different co-stimulatory molecules, including ligands for CD28 and 4-1BB, indicating that they can be modified to modulate T cell stimulation in a controlled manner, promoting expansion of desired T cell subsets [179]. Safety and utility of this cell line was confirmed in several clinical trials. Advances in the development of K562-based aAPCs for T cell expansion were comprehensively reviewed by Butler and Hirano [178].

Currently, the most widely used artificial antigen presenting system is based on magnetic beads coated with anti-CD3 and anti-CD28 antibodies. They were developed by Levine and co-authors and reported to preferentially promote expansion of CD4+ T cells [176]. Anti-CD3/CD28 coated beads provide a simple, consistent and standardised method for T cell activation and expansion. In addition, good manufacturing practice (GMP) grade beads are available [86,180] and this approach meets with greater acceptance of the regulatory bodies than the use of cell-based aAPCs. However, the major disadvantage associated with this system is that beads cannot provide such a variety of co-stimulatory signals as cell-based APCs and have no capability of cytokine release.

Comparison of bead-based and cell-based antigen presenting approaches revealed that anti-CD3/CD28 coated beads are highly effective in CD4+ T cell expansion, whereas irradiated PBMCs coated with anti-CD3 antibodies might be more potent for expansion of CD8+ T cells [181]. However, the underlying mechanism is not fully understood. A potential explanation was provided by Kagotya and co-authors, who demonstrated that CD8+ T cell expansion is significantly enhanced when T cell stimulation is relatively short [182]. Beads are found to persistently stimulate T cells during expansion phase, whereas cell-based aAPCs are readily lysed upon ligation with T cells and their number significantly decreases over time, resulting in a short period of stimulation. Kagotya and co-authors mimicked this transient interaction by removal of anti-CD3/CD28 coated beads after a short period of time. This simple modification of the culture protocol enhanced T cell proliferative potential and resulted in maintenance of Tscm phenotype. Moreover, these results suggest that CD4+ and CD8+ T cells require different time of stimulation for optimal in vitro expansion.

In summary, accumulating data revealed that growth of different T cell subsets can be promoted and their phenotypic qualities can be modulated by manipulating with antigen presenting system and duration of T cell stimulation.

## 12. Improvements in Modulation of Anti-Tumour Potential of T Cells

Attempts are being made to develop strategies which enhance persistence and expansion of adoptively transferred T cells and thus augment the therapeutic effect of ACT, including ex vivo culture conditions, genetic modification of T cells, patient preconditioning and co-infusion with adjuvants.

### 12.1. Lymphodepletion

One of the factors having a crucial impact on persistence and anti-tumour function of adoptively transferred T cells is lymphodepletion preceding the T cell infusion [77]. Chemotherapy with cyclophosphamide and fludarabine is used for elimination of immunosuppressive cells such as Tregs, as well as for elimination of endogenous non-tumour-specific T cells that compete with therapeutic cells for interactions with APCs and activating cytokines, such as IL-7 and IL-15 [183].

### 12.2. Stimulation of T Cells with Cytokines 

Several cytokines have been used to enhance ACT efficacy and were comprehensively reviewed elsewhere [184,185]. The most thoroughly studied examples are described below.

High doses of IL-2 has been used for T cell culture for more than 20 years, and were found to promote growth of tumour reactive T cells. IL-2 is also commonly administered to the patients to enhance proliferation and survival of the transferred TILs. However, this cytokine is essential for development and maintenance of Tregs that can promote tumour progression [186] and stimulates differentiation of T cells into memory subsets [187]. Moreover, IL-2 might cause severe toxicity, when administered in high-doses, yet in lower doses the therapeutic effect is unsatisfactory [188]. This can be at least partially explained by the results of clinical trials concerning autoimmune diseases which demonstrated that low doses of IL-2 specifically activated and expanded Tregs [189,190,191]. Another tested approach was based on adoptive transfer of genetically modified IL-2-secreting TILs, but it also did not increase their in vivo persistence or therapeutic effectiveness [192]. One of the explanations might be the fact that naïve CD8+ T cells do not express α-chain of high-affinity receptor of IL-2 (CD25/ILR2A), while its expression is constitutive for Tregs. Thus, in more recent studies to circumvent this issue, specific mutations were introduced into IL-2 or both IL-2 and its receptor to preferentially stimulate infused CD8+ T cells, but not Tregs. These strategies resulted in high therapeutic efficacy and decreased toxicity in a mouse model [193,194].

IL-2 as the main known mitogen of T cells was the first, but not the only one cytokine tested for cultures of anti-tumour T cells. Caserta at al. reported that IL-7 was superior to IL-2 for expansion of tumour-reactive CD4+ T cells. In their study, cells cultured with IL-7 demonstrated greater anti-tumour potential upon adoptive transfer to tumour-bearing mice [195]. Clinical trials revealed that IL-7 monotherapy resulted in expansion of CD8+ Tc and CD4+ Th cells, but not CD4+ Tregs [196,197]. Recently, Ding and co-authors showed that co-administration of IL-7 with tumour-reactive CD4+ Th cells promoted their expansion, persistence and anti-tumour activity in a mouse model, thus providing a rationale for using IL-7 as an adjuvant for CD4+ Th cell-based adoptive immunotherapy [198].

Recently, IL-15 was shown to be equally effective in promoting T cell expansion as IL-2, but provided higher T cell viability. In preclinical studies, IL-15 administration induced activation, proliferation and survival of T cells without stimulation of Tregs. However, the major limitation of IL-15 is its short in vivo half-life. In consequence, high doses are required for therapeutic effect that leads to toxicity. To address this issue, several different genetic modifications of IL-15 were developed to prolong its half-life, which resulted in an enhanced anti-tumour response in animal models [199]. Currently, clinical trials are ongoing to assess anti-cancer potency of an IL-15 variant called ALT-803. This variant was also tested in a mouse model in combination with ACT with TCR-engineered T cells. Interestingly, for that purpose, a specific cytokine delivery system was applied. This approach was based on T cell surface-conjugated protein nanogels (NGs) that released ALT-803 in response to TCR activation. Treatment of a murine tumour with T cells carrying the ALT-803 resulted in their 16-fold higher expansion as compared to T cells transferred in combination with free ALT-803. Noteworthy, this system enabled delivery of significantly higher doses of cytokine without cytotoxicity and substantially increased tumour regression in a mouse model [200].

IL-18 activates NK and Th cells for IFN-γ production and enhances their anti-tumour activity in cooperation with IL-12. Recently, Kunert at al. compared the effects of IL-12 and IL-18 production by TCR-engineered T cells on murine tumour. Their experiments revealed that T cells harbouring inducible IL-12 had limited anti-tumour effects and severe toxicity in vivo, whereas T cells expressing IL-18 resulted in enhanced anti-tumour responses without toxicity [201]. It provided rationale for further studies on the potential of IL-18 to improve ACT with T cells.

Recently, IL-21 was demonstrated to induce Tscm cell formation from naive CD8+ T cells in vitro [202]. IL-21 was also found to cause greater expansion of less differentiated “young” CD8+ T cells than IL-2 when provided by aAPCs [203].

Although IL-2 is currently the only interleukin approved for clinical use by the Food and Drug Administration (FDA), it is very likely that in the close future cocktails of cytokines will be used for ex vivo expansion and patient treatment in combination with T cell-based therapies, as they promote different biological effects and can act synergistically.

### 12.3. Activating T Cell Co-Stimulatory Receptors 

It was shown that 4-1BB signalling preferentially expands memory CD8+ T cells, whereas CD28 co-stimulation promotes expansion of naive T cells [204]. In addition, anti-4-1BB antibodies were found to preserve CD28 expression in ex vivo expanded CD8+ T cells and enhanced their anti-tumour effector functions [205]. Weigelin et al. also showed that 4-1BB agonistic antibodies improved anti-tumour activity of adoptively transferred CD8+ T cells in a murine melanoma model [206]. Currently, scientists put much effort to reduce toxicity of anti-4-1BB antibodies and maximise their anti-tumour efficacy [207].

Another co-stimulatory receptor that has been tested recently as a potential target of combination therapy with ACT is OX-40 (CD134/TNFRSF4), a member of anti-tumor necrosis factor (TNF) superfamily. Studies on a murine thymoma model revealed that anti-OX40 antibody treatment promoted anti-tumour activity of adoptively transferred CD8^+^ T cells. Interestingly, anti-OX40 antibodies mediated this effect indirectly, by increasing expansion of endogenous CD4^+^ T cells and promoting the interplay between CD4+ and CD8+ T cells [208].

### 12.4. Targeting Immune Checkpoints 

Therapy based on immune checkpoint blockade (ICB) targets T cell inhibitory receptors such as programmed cell death-1 (PD-1) and cytotoxic T lymphocyte antigen-4 (CTLA-4), thus leading to activation of tumour-specific T cells. Anti PD-1/PD-L1 and CTLA-4 inhibitors demonstrated remarkable therapeutic effects and were approved by FDA for treatment of different types of cancer [209,210]. Adoptively transferred T cells may upregulate PD-1 and CTLA-4 upon chronic exposure to tumour antigens, therefore combination of ACT with ICB might be a promising strategy that will improve clinical response rates. Preclinical studies revealed that PD-1 blockade increased the number of transferred T cells at the tumour site and enhanced tumour regression [211]. ACT with MART1-specific T cells combined with CTLA-4 blockade resulted in long-term T cell persistence and durable tumour regression in metastatic melanoma patients [212]. Dual blockade of CTLA-4 and PD-1 enhanced adoptive T-cell therapy efficacy in a murine melanoma model [213]. Currently, several clinical trials are investigating the combination of ACT with TILs or TCR-engineered T cells and ICB antibodies [214].

### 12.5. T Cell Therapies in Combination with Dendritic Cell-Based Vaccines

Treatment with dendritic cell-based vaccine followed by ACT with TILs resulted in complete remission in one melanoma patient and stable disease in two others [215]. Clinical trials with TCR gene modified T cells and peptide-pulsed dendritic cells demonstrated transient tumour responses in melanoma patients, emphasising the need for protocol improvement in this therapeutic approach [216,217].

### 12.6. Enhancing Trafficking of T Cells to Solid Tumour Sites

Prerequisite for effective ACT treatment is trafficking of transferred T cells to the tumour site. Therefore, strategies for improvement of T cell migration to tumour sites are being developed. One of these approaches utilises the fact that cancer cells secrete specific chemokines, which attract cells that express matching chemokine receptors. For instance, melanoma cells produce CXCL1 and CXCL8 chemokines, which are ligands for CXCR2 receptor. Studies on animal models indicated that transfer of T cells genetically modified to express CXCR2 enhanced their homing to the tumour site and improved anti-tumour immune response [218,219]. Currently, therapeutic effectiveness of this strategy is being tested in an ongoing clinical trial.

### 12.7. Targeting T Cell Metabolism to Boost Their Anti-Tumour Potential

Interesting strategies aimed at reprogramming T cell metabolism were developed to potentiate the activity of transferred T cells. Ho and co-authors showed that glycolytic metabolite, phosphoenolpyruvate (PEP), serves as a sensor for glucose availability and is critical for controlling T cell function. Metabolic reprogramming of T cells to increase PEP production in low-glucose tumour microenvironment improved their anti-tumour responses [220]. In another study metabolic reprogramming of T cells via enforced expression of PGC1α (peroxisome proliferator-activated receptor gamma co-activator 1-alpha) rescued mitochondrial function of T cells and enhanced their anti-tumour activity [221]. Development of approaches that modify metabolism of T cells in the TME holds promise for improvement of ACT efficacy. Recent advances in understanding of the role of T cell metabolic pathways in regulation of their function and interplay between cancer cells and T cells in TME have been recently reviewed in detail [222,223].

## 13. Conclusions

The remarkable success of CAR T cell therapy in the treatment of refractor pre-B cell acute lymphoblastic leukaemia and diffuse large B cell lymphoma led to an intensive research on the possible use of this kind of therapy for solid tumours. However, the major limitation of CAR T cells is that they can only recognise cell surface proteins. Since surface tumour-specific antigens are scarce, CAR T cells may target almost exclusively TAAs, posing a risk of cross-reactivity with normal tissues and leading to serious safety concerns.

Although intensive efforts are made to overcome limited cancer-specificity of CAR T cells, TCR therapy may be a more promising alternative for targeting solid tumours. TCR signalling confers recognition of much wider repertoire of tumour antigens and recent studies revealed that neoantigen-specific T cells can be detected in a variety of solid tumours, regardless of their mutational load.

**Figure 2:**
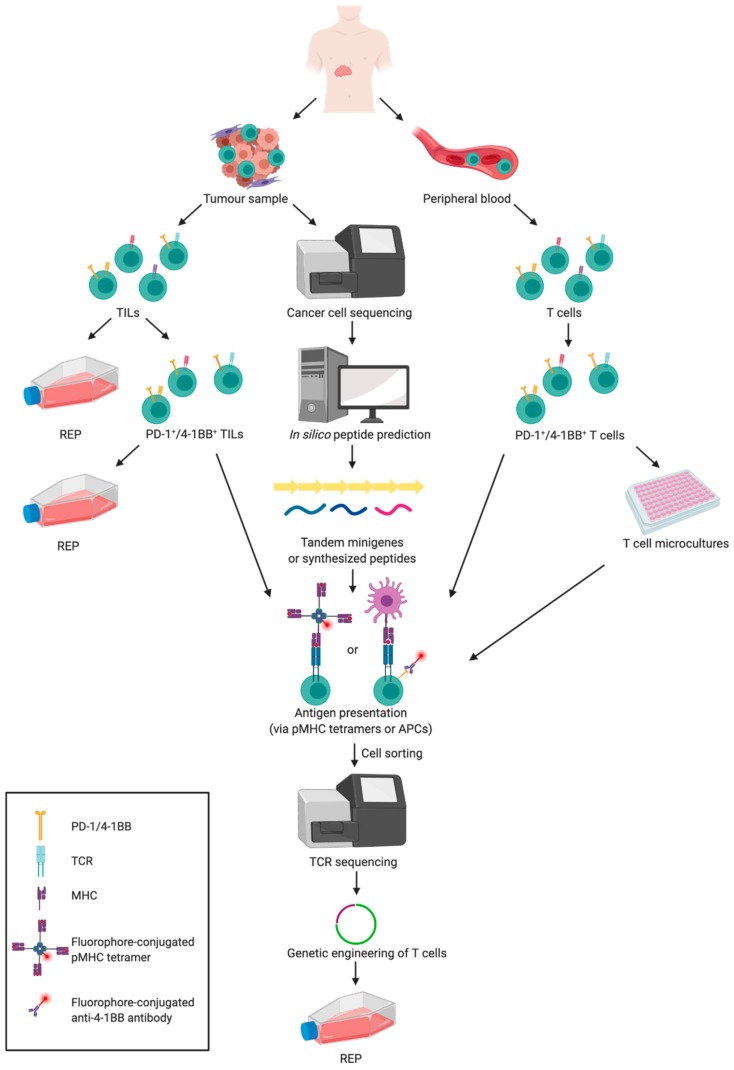
Strategies for T cell selection for adoptive cell therapy (ACT) of cancer. Resected tumour and peripheral blood samples are two main sources of T cells for ACT of cancer. Tumour samples require processing to obtain tumour infiltrating lymphocytes (TILs). Heterogenous TIL population can be expanded according to the rapid expansion protocol (REP). Alternatively, TILs expressing programmed death receptor 1 (PD-1) or 4-1BB can be selected, to increase frequency of tumour-specific T cells, which are subsequently expanded. Another approach includes cancer cell sequencing followed by in silico peptide prediction and tandem minigenes (TMGs) or synthetic peptide generation. Subsequently, T cells isolated from cancer patient, after optional enrichment for PD-1^+^/4-1BB^+^ cells, are tested for reactivity to predicted neopeptides with one of the following approaches: binding to MHC tetramers loaded with synthetic peptides (pMHC tetramers); and expression of activation marker (e.g., 4-1BB) in the presence of APCs transfected with TMGs or pulsed with synthetic peptides. Then, antigen-specific T cells are sorted and their TCRs are sequenced in aim to introduce tumour-specific TCRs into polyclonal T cells. Subsequently, TCR-engineered T cells are expanded for therapeutically relevant numbers (the figure was created with BioRender).

TCR therapy was first used 30 years ago but since then many of its aspects have been developed. Methods for enrichment of tumour-reactive TILs evolved from culture of T cells in the presence of cancer cells through selection of activated or PD-1-expressing T cells to isolation of T cells specific for the defined antigen (with a recent focus on neoantigens). Once the neopeptides are identified, they have to be analysed for their immunogenicity either with the use of standard T cell activity assays or more sophisticated methods (e.g., pMHC multimers). Finally, the selected T cells have to be expanded, or alternatively a specific TCR can be introduced into polyclonal T cells via a viral vector or CRISPR technology. Phenotypically different types of T cells have been tested to ensure efficient and durable anti-tumour responses. Recent approaches also involve the use of tumour-specific T cells isolated from peripheral blood of patients. The overview of the past and current strategies of T cell selection for anti-cancer therapies is presented in Figure 2.

The future studies will presumably focus on the development of TCR therapies targeting hot-spot driver mutation-derived neoantigens. Several neoantigens of this kind have been discovered very recently (e.g., the ones derived from KRAS G12D [123,136] or p53 hot spot mutations [125,139,140]), raising the possibility of generating “off-the-shelf ” TCR-engineered T cell products for MHC-matched patients with tumours expressing these neoantigens.

However, the major limitation of TCR therapies is the prerequisite of MHC expression by the tumour. It is well known that downregulation or total loss of MHC expression is one of the main mechanisms of tumour immune evasion [1]. Nevertheless, harnessing natural killer (NK) cells may hold a promise to circumvent this issue, as they can eliminate MHC-deficient cancer cells, thus complementing anti-tumour activity of T cells [224,225]. An exciting prospect for efficient adoptive cell therapy would be infusion of both NK and T cells. Alternatively, TCR therapy could be combined with additives that protect both T and NK cells from tumour mediated immunosuppression such as immune checkpoint inhibitors (e.g., anti-PD-1 antibodies) or cytokines (e.g., IL-15). Therefore, in the future, great efforts will probably be made to harness our current understanding of T cell biology, TCR antigen recognition mechanisms and immune response regulation into the design of combination therapy against solid tumours.

## Figures and Tables

**Table 1:** Strategies for improvement of Chimeric Antigen Receptor (CAR) T cell therapies for solid tumours.

Challenge	Possible Solution	References
chimeric antigen receptor (CAR) T cell migration to the tumour site	Introduction of C-C chemokine receptor type 2 (CCR2) into CAR T cell—chemotaxis toward CC motif chemokine ligand 2 (CCL2) secreted by cancer cells	[27,28]
	Development of CAR targeting fibroblast activation protein (FAP) expressed on immunosuppressive stromal cells often associated with epithelial tumours	[29,30]
	Local CAR T cell administration (i.e., intratumoural injections)	[31,32]
Limited in vivo persistence and proliferation of CAR T cells	Using distinct co-stimulatory molecules for CD4+ and CD8+ T cell subsets (i.e., CD4.ICOS-CAR T cells and CD8.41BB-CAR T cells)	[33]
	Addition of a second, independent co-stimulatory molecule, i.e., 4-1BB ligand, CD40L	[34,35]
	Addition of inducible MyD88/CD40 (iMC), to activate downstream toll-like receptor (TLR) and CD40 signalling pathways using a small molecule ligand, rimiducid	[36,37]
	Constitutive expression of a cytokine that is bound to cell membrane of CAR T cell or secreted by the cell, i.e., interleukin (IL) 15, IL-12	[38,39,40]
	Constitutive activation of intracellular IL-7 cytokine receptor triggering IL-7 axis without stimulating bystander lymphocytes	[41]
Limited tumour specificity and off-target effects	The use of combinatorial antigen sensing circuits, i.e., synthetic Notch, where engagement of a tissue-specific antigen by a surface receptor induces transcription of a CAR recognising a tumour-associated antigen	[42,43,44]
	Reduction of CAR T cell affinity	[45]
	Designing CAR T cells targeting antigens that contain tumour-specific modifications/mutations (i.e., mutated variant III of epidermal growth factor receptor; EGFRvIII)	[46,47,48]
Overcoming immunosuppressive tumour microenvironment (TME)	CAR T cell transduction with dominant-negative transforming growth factor β receptor II (dnTGF-βRII)—a decoy receptor for immunosuppressive TGFβ produced by tumour	[49]
	Introducing switch receptors transforming inhibitory cytokine signals into a stimulus (i.e., fusing immunosuppressive IL-4 receptor exodomain to the immunostimulatory IL-7 receptor endodomain)	[50,51]
	Introduction of hypoxia-inducible factor 1-alpha (HIF-1α), a transcription factor stabilised in response to hypoxia, to CAR T cells resulting in increased CAR expression specifically in hypoxic TME	[52]
	Co-expression of catalase to protect CAR T cells, as well as bystander T cells, from reactive oxygen species (ROS) in TME	[53]
	Inhibition of adenosine receptors with their antagonists or shRNA to prevent immunosuppressive effects exerted by tumour derived adenosine	[54]

## References

[1] Garrido F., Aptsiauri N., Doorduijn E.M., Garcia Lora A.M., van Hall T. (2016). The urgent need to recover MHC class I in cancers for effective immunotherapy. Curr. Opin. Immunol..

[2] Hochweller K., Wabnitz G.H., Samstag Y., Suffner J., Hämmerling G.J., Garbi N. (2010). Dendritic cells control T cell tonic signaling required for responsiveness to foreign antigen. Proc. Natl. Acad. Sci. USA.

[3] Birnbaum M.E., Mendoza J.L., Sethi D.K., Dong S., Glanville J., Dobbins J., Ozkan E., Davis M.M., Wucherpfennig K.W., Garcia K.C. (2014). Deconstructing the peptide-MHC specificity of T cell recognition. Cell.

[4] Gaud G., Lesourne R., Love P.E. (2018). Regulatory mechanisms in T cell receptor signalling. Nat. Rev. Immunol..

[5] Esin S., Shigematsu M., Nagai S., Eklund A., Wigzell H., Grunewald J. (1996). Different percentages of peripheral blood gamma delta + T cells in healthy individuals from different areas of the world. Scand. J. Immunol..

[6] Garcillán B., Marin A.V., Jiménez-Reinoso A., Briones A.C., Muñoz-Ruiz M., García-León M.J., Gil J., Allende L.M., Martínez-Naves E., Toribio M.L. (2015). γδ T Lymphocytes in the Diagnosis of Human T Cell Receptor Immunodeficiencies. Front. Immunol..

[7] Harris D.T., Kranz D.M. (2016). Adoptive T Cell Therapies: A Comparison of T Cell Receptors and Chimeric Antigen Receptors. Trends Pharmacol. Sci..

[8] Artyomov M.N., Lis M., Devadas S., Davis M.M., Chakraborty A.K. (2010). CD4 and CD8 binding to MHC molecules primarily acts to enhance Lck delivery. Proc. Natl. Acad. Sci. USA.

[9] Greenwald R.J., Freeman G.J., Sharpe A.H. (2005). The B7 family revisited. Annu. Rev. Immunol..

[10] Croft M., So T., Duan W., Soroosh P. (2009). The significance of OX40 and OX40L to T-cell biology and immune disease. Immunol. Rev..

[11] Sanchez-Paulete A.R., Labiano S., Rodriguez-Ruiz M.E., Azpilikueta A., Etxeberria I., Bolaños E., Lang V., Rodriguez M., Aznar M.A., Jure-Kunkel M. (2016). Deciphering CD137 (4-1BB) signaling in T-cell costimulation for translation into successful cancer immunotherapy. Eur. J. Immunol..

[12] Wikenheiser D.J., Stumhofer J.S. (2016). ICOS Co-Stimulation: Friend or Foe?. Front. Immunol..

[13] Pardoll D.M. (2012). The blockade of immune checkpoints in cancer immunotherapy. Nat. Rev. Cancer.

[14] June C.H., O’Connor R.S., Kawalekar O.U., Ghassemi S., Milone M.C. (2018). CAR T cell immunotherapy for human cancer. Science.

[15] Chandran S.S., Klebanoff C.A. (2019). T cell receptor-based cancer immunotherapy: Emerging efficacy and pathways of resistance. Immunol. Rev..

[16] Ying Z., Huang X.F., Xiang X., Liu Y., Kang X., Song Y., Guo X., Liu H., Ding N., Zhang T. (2019). A safe and potent anti-CD19 CAR T cell therapy. Nat. Med..

[17] Zheng P.P., Kros J.M., Li J. (2018). Approved CAR T cell therapies: ice bucket challenges on glaring safety risks and long-term impacts. Drug Discov. Today.

[18] Friedman K.M., Garrett T.E., Evans J.W., Horton H.M., Latimer H.J., Seidel S.L., Horvath C.J., Morgan R.A. (2018). Effective Targeting of Multiple B-Cell Maturation Antigen-Expressing Hematological Malignances by Anti-B-Cell Maturation Antigen Chimeric Antigen Receptor T Cells. Hum. Gene. Ther..

[19] Smith E.L., Staehr M., Masakayan R., Tatake I.J., Purdon T.J., Wang X., Wang P., Liu H., Xu Y., Garrett-Thomson S.C. (2018). Development and Evaluation of an Optimal Human Single-Chain Variable Fragment-Derived BCMA-Targeted CAR T Cell Vector. Mol. Ther..

[20] Stamenkovic I., Seed B. (1988). CD19, the earliest differentiation antigen of the B cell lineage, bears three extracellular immunoglobulin-like domains and an Epstein-Barr virus-related cytoplasmic tail. J. Exp. Med..

[21] Wang K., Wei G., Liu D. (2012). CD19: a biomarker for B cell development, lymphoma diagnosis and therapy. Exp. Hematol. Oncol..

[22] Watanabe K., Kuramitsu S., Posey A.D., June C.H. (2018). Expanding the Therapeutic Window for CAR T Cell Therapy in Solid Tumors: The Knowns and Unknowns of CAR T Cell Biology. Front. Immunol..

[23] DeRenzo C., Gottschalk S. (2019). Genetic Modification Strategies to Enhance CAR T Cell Persistence for Patients With Solid Tumors. Front. Immunol..

[24] Knochelmann H.M., Smith A.S., Dwyer C.J., Wyatt M.M., Mehrotra S., Paulos C.M. (2018). CAR T Cells in Solid Tumors: Blueprints for Building Effective Therapies. Front. Immunol..

[25] Martinez M., Moon E.K. (2019). CAR T Cells for Solid Tumors: New Strategies for Finding, Infiltrating, and Surviving in the Tumor Microenvironment. Front. Immunol..

[26] Mirzaei H.R., Rodriguez A., Shepphird J., Brown C.E., Badie B. (2017). Chimeric Antigen Receptors T Cell Therapy in Solid Tumor: Challenges and Clinical Applications. Front. Immunol..

[27] Craddock J.A., Lu A., Bear A., Pule M., Brenner M.K., Rooney C.M., Foster A.E. (2010). Enhanced tumor trafficking of GD2 chimeric antigen receptor T cells by expression of the chemokine receptor CCR2b. J. Immunother..

[28] Moon E.K., Carpenito C., Sun J., Wang L.C., Kapoor V., Predina J., Powell D.J., Riley J.L., June C.H., Albelda S.M. (2011). Expression of a functional CCR2 receptor enhances tumor localization and tumor eradication by retargeted human T cells expressing a mesothelin-specific chimeric antibody receptor. Clin. Cancer Res..

[29] Schuberth P.C., Hagedorn C., Jensen S.M., Gulati P., van den Broek M., Mischo A., Soltermann A., Jüngel A., Marroquin Belaunzaran O., Stahel R. (2013). Treatment of malignant pleural mesothelioma by fibroblast activation protein-specific re-directed T cells. J. Transl. Med..

[30] Tran E., Chinnasamy D., Yu Z., Morgan R.A., Lee C.C., Restifo N.P., Rosenberg S.A. (2013). Immune targeting of fibroblast activation protein triggers recognition of multipotent bone marrow stromal cells and cachexia. J. Exp. Med..

[31] Priceman S.J., Tilakawardane D., Jeang B., Aguilar B., Murad J.P., Park A.K., Chang W.C., Ostberg J.R., Neman J., Jandial R. (2018). Regional Delivery of Chimeric Antigen Receptor-Engineered T Cells Effectively Targets HER2. Clin. Cancer Res..

[32] Tchou J., Zhao Y., Levine B.L., Zhang P.J., Davis M.M., Melenhorst J.J., Kulikovskaya I., Brennan A.L., Liu X., Lacey S.F. (2017). Safety and Efficacy of Intratumoral Injections of Chimeric Antigen Receptor (CAR) T Cells in Metastatic Breast Cancer. Cancer Immunol. Res..

[33] Guedan S., Posey A.D., Shaw C., Wing A., Da T., Patel P.R., McGettigan S.E., Casado-Medrano V., Kawalekar O.U., Uribe-Herranz M. (2018). Enhancing CAR T cell persistence through ICOS and 4-1BB costimulation. JCI Insight..

[34] Zhao Z., Condomines M., van der Stegen S.J.C., Perna F., Kloss C.C., Gunset G., Plotkin J., Sadelain M. (2015). Structural Design of Engineered Costimulation Determines Tumor Rejection Kinetics and Persistence of CAR T Cells. Cancer Cell.

[35] Curran K.J., Seinstra B.A., Nikhamin Y., Yeh R., Usachenko Y., van Leeuwen D.G., Purdon T., Pegram H.J., Brentjens R.J. (2015). Enhancing antitumor efficacy of chimeric antigen receptor T cells through constitutive CD40L expression. Mol. Ther..

[36] Foster A.E., Mahendravada A., Shinners N.P., Chang W.C., Crisostomo J., Lu A., Khalil M., Morschl E., Shaw J.L., Saha S. (2017). Regulated Expansion and Survival of Chimeric Antigen Receptor-Modified T Cells Using Small Molecule-Dependent Inducible MyD88/CD40. Mol. Ther..

[37] Mata M., Gerken C., Nguyen P., Krenciute G., Spencer D.M., Gottschalk S. (2017). Inducible Activation of MyD88 and CD40 in CAR T Cells Results in Controllable and Potent Antitumor Activity in Preclinical Solid Tumor Models. Cancer Discov..

[38] Hurton L.V., Singh H., Najjar A.M., Switzer K.C., Mi T., Maiti S., Olivares S., Rabinovich B., Huls H., Forget M.A. (2016). Tethered IL-15 augments antitumor activity and promotes a stem-cell memory subset in tumor-specific T cells. Proc. Natl. Acad. Sci. USA.

[39] Krenciute G., Prinzing B.L., Yi Z., Wu M.F., Liu H., Dotti G., Balyasnikova I.V., Gottschalk S. (2017). Transgenic Expression of IL15 Improves Antiglioma Activity of IL13Rα2-CAR T Cells but Results in Antigen Loss Variants. Cancer Immunol. Res..

[40] Koneru M., Purdon T.J., Spriggs D., Koneru S., Brentjens R.J. (2015). IL-12 secreting tumor-targeted chimeric antigen receptor T cells eradicate ovarian tumors. Oncoimmunology.

[41] Shum T., Omer B., Tashiro H., Kruse R.L., Wagner D.L., Parikh K., Yi Z., Sauer T., Liu D., Parihar R. (2017). Constitutive Signaling from an Engineered IL7 Receptor Promotes Durable Tumor Elimination by Tumor-Redirected T Cells. Cancer Discov..

[42] Morsut L., Roybal K.T., Xiong X., Gordley R.M., Coyle S.M., Thomson M., Lim W.A. (2016). Engineering Customized Cell Sensing and Response Behaviors Using Synthetic Notch Receptors. Cell.

[43] Roybal K.T., Williams J.Z., Morsut L., Rupp L.J., Kolinko I., Choe J.H., Walker W.J., McNally K.A., Lim W.A. (2016). Engineering T Cells with Customized Therapeutic Response Programs Using Synthetic Notch Receptors. Cell.

[44] Roybal K.T., Rupp L.J., Morsut L., Walker W.J., McNally K.A., Park J.S., Lim W.A. (2016). Precision Tumor Recognition by T Cells With Combinatorial Antigen-Sensing Circuits. Cell.

[45] Park S., Shevlin E., Vedvyas Y., Zaman M., Hsu Y.S., Min I.M., Jin M.M. (2017). Micromolar affinity CAR T cells to ICAM-1 achieves rapid tumor elimination while avoiding systemic toxicity. Sci. Rep..

[46] Johnson L.A., Scholler J., Ohkuri T., Kosaka A., Patel P.R., McGettigan S.E., Nace A.K., Dentchev T., Thekkat P., Loew A. (2015). Rational development and characterization of humanized anti-EGFR variant III chimeric antigen receptor T cells for glioblastoma. Sci. Transl. Med..

[47] O’Rourke D.M., Nasrallah M.P., Desai A., Melenhorst J.J., Mansfield K., Morrissette J.J.D., Martinez-Lage M., Brem S., Maloney E., Shen A. (2017). A single dose of peripherally infused EGFRvIII-directed CAR T cells mediates antigen loss and induces adaptive resistance in patients with recurrent glioblastoma. Sci. Transl. Med..

[48] Posey A.D., Schwab R.D., Boesteanu A.C., Steentoft C., Mandel U., Engels B., Stone J.D., Madsen T.D., Schreiber K., Haines K.M. (2016). Engineered CAR T Cells Targeting the Cancer-Associated Tn-Glycoform of the Membrane Mucin MUC1 Control Adenocarcinoma. Immunity.

[49] Kloss C.C., Lee J., Zhang A., Chen F., Melenhorst J.J., Lacey S.F., Maus M.V., Fraietta J.A., Zhao Y., June C.H. (2018). Dominant-Negative TGF-β Receptor Enhances PSMA-Targeted Human CAR T Cell Proliferation And Augments Prostate Cancer Eradication. Mol. Ther..

[50] Mohammed S., Sukumaran S., Bajgain P., Watanabe N., Heslop H.E., Rooney C.M., Brenner M.K., Fisher W.E., Leen A.M., Vera J.F. (2017). Improving Chimeric Antigen Receptor-Modified T Cell Function by Reversing the Immunosuppressive Tumor Microenvironment of Pancreatic Cancer. Mol. Ther..

[51] Bajgain P., Tawinwung S., D’Elia L., Sukumaran S., Watanabe N., Hoyos V., Lulla P., Brenner M.K., Leen A.M., Vera J.F. (2018). CAR T cell therapy for breast cancer: harnessing the tumor milieu to drive T cell activation. J. Immunother. Cancer.

[52] Juillerat A., Marechal A., Filhol J.M., Valogne Y., Valton J., Duclert A., Duchateau P., Poirot L. (2017). An oxygen sensitive self-decision making engineered CAR T-cell. Sci. Rep..

[53] Ligtenberg M.A., Mougiakakos D., Mukhopadhyay M., Witt K., Lladser A., Chmielewski M., Riet T., Abken H., Kiessling R. (2016). Coexpressed Catalase Protects Chimeric Antigen Receptor-Redirected T Cells as well as Bystander Cells from Oxidative Stress-Induced Loss of Antitumor Activity. J. Immunol..

[54] Beavis P.A., Henderson M.A., Giuffrida L., Mills J.K., Sek K., Cross R.S., Davenport A.J., John L.B., Mardiana S., Slaney C.Y. (2017). Targeting the adenosine 2A receptor enhances chimeric antigen receptor T cell efficacy. J. Clin. Investig..

[55] Neelapu S.S., Tummala S., Kebriaei P., Wierda W., Gutierrez C., Locke F.L., Komanduri K.V., Lin Y., Jain N., Daver N. (2018). Chimeric antigen receptor T-cell therapy - assessment and management of toxicities. Nat. Rev. Clin. Oncol..

[56] Di Stasi A., Tey S.K., Dotti G., Fujita Y., Kennedy-Nasser A., Martinez C., Straathof K., Liu E., Durett A.G., Grilley B. (2011). Inducible apoptosis as a safety switch for adoptive cell therapy. N. Engl. J. Med..

[57] Bielamowicz K., Fousek K., Byrd T.T., Samaha H., Mukherjee M., Aware N., Wu M.F., Orange J.S., Sumazin P., Man T.K. (2018). Trivalent CAR T cells overcome interpatient antigenic variability in glioblastoma. Neuro Oncol..

[58] Caruso H., Heimberger A.B. (2018). Comment on “Trivalent CAR T cells overcome interpatient antigenic variability in glioblastoma”. Neuro Oncol..

[59] Morgan R.A., Yang J.C., Kitano M., Dudley M.E., Laurencot C.M., Rosenberg S.A. (2010). Case report of a serious adverse event following the administration of T cells transduced with a chimeric antigen receptor recognizing ERBB2. Mol. Ther..

[60] Richman S.A., Nunez-Cruz S., Moghimi B., Li L.Z., Gershenson Z.T., Mourelatos Z., Barrett D.M., Grupp S.A., Milone M.C. (2018). High-Affinity GD2-Specific CAR T Cells Induce Fatal Encephalitis in a Preclinical Neuroblastoma Model. Cancer Immunol. Res..

[61] Bonifant C.L., Jackson H.J., Brentjens R.J., Curran K.J. (2016). Toxicity and management in CAR T-cell therapy. Mol. Ther. Oncolytics..

[62] Morgan R.A., Chinnasamy N., Abate-Daga D., Gros A., Robbins P.F., Zheng Z., Dudley M.E., Feldman S.A., Yang J.C., Sherry R.M. (2013). Cancer regression and neurological toxicity following anti-MAGE-A3 TCR gene therapy. J. Immunother..

[63] Wagner S., Mullins C.S., Linnebacher M. (2018). Colorectal cancer vaccines: Tumor-associated antigens. World J. Gastroenterol..

[64] Jiang T., Shi T., Zhang H., Hu J., Song Y., Wei J., Ren S., Zhou C. (2019). Tumor neoantigens: from basic research to clinical applications. J. Hematol. Oncol..

[65] Zamora A.E., Crawford J.C., Thomas P.G. (2018). Hitting the Target: How T Cells Detect and Eliminate Tumors. J. Immunol..

[66] Lawrence M.S., Stojanov P., Polak P., Kryukov G.V., Cibulskis K., Sivachenko A., Carter S.L., Stewart C., Mermel C.H., Roberts S.A. (2013). Mutational heterogeneity in cancer and the search for new cancer-associated genes. Nature.

[67] Zhang J., Wang L. (2019). The Emerging World of TCR-T Cell Trials Against Cancer: A Systematic Review. Technol. Cancer Res. Treat..

[68] Ott P.A., Hu Z., Keskin D.B., Shukla S.A., Sun J., Bozym D.J., Zhang W., Luoma A., Giobbie-Hurder A., Peter L. (2017). An immunogenic personal neoantigen vaccine for patients with melanoma. Nature.

[69] Chalmers Z.R., Connelly C.F., Fabrizio D., Gay L., Ali S.M., Ennis R., Schrock A., Campbell B., Shlien A., Chmielecki J. (2017). Analysis of 100,000 human cancer genomes reveals the landscape of tumor mutational burden. Genome Med..

[70] Vogelstein B., Papadopoulos N., Velculescu V.E., Zhou S., Diaz L.A., Kinzler K.W. (2013). Cancer genome landscapes. Science.

[71] Shen L., Zhang J., Lee H., Batista M.T., Johnston S.A. (2019). RNA Transcription and Splicing Errors as a Source of Cancer Frameshift Neoantigens for Vaccines. Sci. Rep..

[72] Timmers M., Roex G., Wang Y., Campillo-Davo D., Van Tendeloo V.F.I., Chu Y., Berneman Z.N., Luo F., Van Acker H.H., Anguille S. (2019). Chimeric Antigen Receptor-Modified T Cell Therapy in Multiple Myeloma: Beyond B Cell Maturation Antigen. Front. Immunol..

[73] Stone J.D., Harris D.T., Soto C.M., Chervin A.S., Aggen D.H., Roy E.J., Kranz D.M. (2014). A novel T cell receptor single-chain signaling complex mediates antigen-specific T cell activity and tumor control. Cancer Immunol. Immunother..

[74] Xu Y., Yang Z., Horan L.H., Zhang P., Liu L., Zimdahl B., Green S., Lu J., Morales J.F., Barrett D.M. (2018). A novel antibody-TCR (AbTCR) platform combines Fab-based antigen recognition with gamma/delta-TCR signaling to facilitate T-cell cytotoxicity with low cytokine release. Cell Discov..

[75] Rosenberg S.A., Packard B.S., Aebersold P.M., Solomon D., Topalian S.L., Toy S.T., Simon P., Lotze M.T., Yang J.C., Seipp C.A. (1988). Use of tumor-infiltrating lymphocytes and interleukin-2 in the immunotherapy of patients with metastatic melanoma. A preliminary report. N. Engl. J. Med..

[76] Rosenberg S.A., Yannelli J.R., Yang J.C., Topalian S.L., Schwartzentruber D.J., Weber J.S., Parkinson D.R., Seipp C.A., Einhorn J.H., White D.E. (1994). Treatment of patients with metastatic melanoma with autologous tumor-infiltrating lymphocytes and interleukin 2. J. Natl. Cancer. Inst..

[77] Dudley M.E., Wunderlich J.R., Robbins P.F., Yang J.C., Hwu P., Schwartzentruber D.J., Topalian S.L., Sherry R., Restifo N.P., Hubicki A.M. (2002). Cancer regression and autoimmunity in patients after clonal repopulation with antitumor lymphocytes. Science.

[78] Dudley M.E., Wunderlich J.R., Shelton T.E., Even J., Rosenberg S.A. (2003). Generation of tumor-infiltrating lymphocyte cultures for use in adoptive transfer therapy for melanoma patients. J. Immunother..

[79] Dudley M.E., Wunderlich J.R., Yang J.C., Sherry R.M., Topalian S.L., Restifo N.P., Royal R.E., Kammula U., White D.E., Mavroukakis S.A. (2005). Adoptive cell transfer therapy following non-myeloablative but lymphodepleting chemotherapy for the treatment of patients with refractory metastatic melanoma. J. Clin. Oncol..

[80] Rosenberg S.A., Yang J.C., Sherry R.M., Kammula U.S., Hughes M.S., Phan G.Q., Citrin D.E., Restifo N.P., Robbins P.F., Wunderlich J.R. (2011). Durable complete responses in heavily pretreated patients with metastatic melanoma using T-cell transfer immunotherapy. Clin. Cancer Res..

[81] Tran K.Q., Zhou J., Durflinger K.H., Langhan M.M., Shelton T.E., Wunderlich J.R., Robbins P.F., Rosenberg S.A., Dudley M.E. (2008). Minimally cultured tumor-infiltrating lymphocytes display optimal characteristics for adoptive cell therapy. J. Immunother..

[82] Dudley M.E., Gross C.A., Langhan M.M., Garcia M.R., Sherry R.M., Yang J.C., Phan G.Q., Kammula U.S., Hughes M.S., Citrin D.E. (2010). CD8+ enriched "young" tumor infiltrating lymphocytes can mediate regression of metastatic melanoma. Clin. Cancer Res..

[83] Prieto P.A., Durflinger K.H., Wunderlich J.R., Rosenberg S.A., Dudley M.E. (2010). Enrichment of CD8+ cells from melanoma tumor-infiltrating lymphocyte cultures reveals tumor reactivity for use in adoptive cell therapy. J. Immunother..

[84] Shimizu J., Yamazaki S., Sakaguchi S. (1999). Induction of tumor immunity by removing CD25+CD4+ T cells: a common basis between tumor immunity and autoimmunity. J. Immunol..

[85] Darrasse-Jèze G., Podsypanina K. (2013). How numbers, nature, and immune status of foxp3(+) regulatory T-cells shape the early immunological events in tumor development. Front. Immunol..

[86] Marek-Trzonkowska N., Piekarska K., Filipowicz N., Piotrowski A., Gucwa M., Vogt K., Sawitzki B., Siebert J., Trzonkowski P. (2017). Mild hypothermia provides Treg stability. Sci. Rep..

[87] Marek N., Bieniaszewska M., Krzystyniak A., Juścińska J., Myśliwska J., Witkowski P., Hellmann A., Trzonkowski P. (2011). The time is crucial for ex vivo expansion of T regulatory cells for therapy. Cell Transplant..

[88] Itzhaki O., Hovav E., Ziporen Y., Levy D., Kubi A., Zikich D., Hershkovitz L., Treves A.J., Shalmon B., Zippel D. (2011). Establishment and large-scale expansion of minimally cultured "young" tumor infiltrating lymphocytes for adoptive transfer therapy. J. Immunother..

[89] Besser M.J., Shapira-Frommer R., Treves A.J., Zippel D., Itzhaki O., Schallmach E., Kubi A., Shalmon B., Hardan I., Catane R. (2009). Minimally cultured or selected autologous tumor-infiltrating lymphocytes after a lympho-depleting chemotherapy regimen in metastatic melanoma patients. J. Immunother..

[90] Pilon-Thomas S., Kuhn L., Ellwanger S., Janssen W., Royster E., Marzban S., Kudchadkar R., Zager J., Gibney G., Sondak V.K. (2012). Efficacy of adoptive cell transfer of tumor-infiltrating lymphocytes after lymphopenia induction for metastatic melanoma. J. Immunother..

[91] Radvanyi L.G., Bernatchez C., Zhang M., Fox P.S., Miller P., Chacon J., Wu R., Lizee G., Mahoney S., Alvarado G. (2012). Specific lymphocyte subsets predict response to adoptive cell therapy using expanded autologous tumor-infiltrating lymphocytes in metastatic melanoma patients. Clin. Cancer Res..

[92] Andersen R., Donia M., Ellebaek E., Borch T.H., Kongsted P., Iversen T.Z., Hölmich L.R., Hendel H.W., Met Ö., Andersen M.H. (2016). Long-Lasting Complete Responses in Patients with Metastatic Melanoma after Adoptive Cell Therapy with Tumor-Infiltrating Lymphocytes and an Attenuated IL2 Regimen. Clin. Cancer Res..

[93] Mullinax J.E., Hall M., Prabhakaran S., Weber J., Khushalani N., Eroglu Z., Brohl A.S., Markowitz J., Royster E., Richards A. (2018). Combination of Ipilimumab and Adoptive Cell Therapy with Tumor-Infiltrating Lymphocytes for Patients with Metastatic Melanoma. Front. Oncol..

[94] Pedersen M., Westergaard M.C.W., Milne K., Nielsen M., Borch T.H., Poulsen L.G., Hendel H.W., Kennedy M., Briggs G., Ledoux S. (2018). Adoptive cell therapy with tumor-infiltrating lymphocytes in patients with metastatic ovarian cancer: a pilot study. Oncoimmunology.

[95] Westergaard M.C.W., Andersen R., Chong C., Kjeldsen J.W., Pedersen M., Friese C., Hasselager T., Lajer H., Coukos G., Bassani-Sternberg M. (2019). Tumour-reactive T cell subsets in the microenvironment of ovarian cancer. Br. J. Cancer.

[96] Andersen R., Westergaard M.C.W., Kjeldsen J.W., Müller A., Pedersen N.W., Hadrup S.R., Met Ö., Seliger B., Kromann-Andersen B., Hasselager T. (2018). T-cell Responses in the Microenvironment of Primary Renal Cell Carcinoma-Implications for Adoptive Cell Therapy. Cancer Immunol. Res..

[97] Turcotte S., Gros A., Hogan K., Tran E., Hinrichs C.S., Wunderlich J.R., Dudley M.E., Rosenberg S.A. (2013). Phenotype and function of T cells infiltrating visceral metastases from gastrointestinal cancers and melanoma: Implications for adoptive cell transfer therapy. J. Immunol..

[98] Wolfl M., Kuball J., Ho W.Y., Nguyen H., Manley T.J., Bleakley M., Greenberg P.D. (2007). Activation-induced expression of CD137 permits detection, isolation, and expansion of the full repertoire of CD8+ T cells responding to antigen without requiring knowledge of epitope specificities. Blood.

[99] Ye Q., Song D.G., Poussin M., Yamamoto T., Best A., Li C., Coukos G., Powell D.J. (2014). CD137 accurately identifies and enriches for naturally occurring tumor-reactive T cells in tumor. Clin. Cancer Res..

[100] Seliktar-Ofir S., Merhavi-Shoham E., Itzhaki O., Yunger S., Markel G., Schachter J., Besser M.J. (2017). Selection of Shared and Neoantigen-Reactive T Cells for Adoptive Cell Therapy Based on CD137 Separation. Front. Immunol..

[101] Inozume T., Hanada K., Wang Q.J., Ahmadzadeh M., Wunderlich J.R., Rosenberg S.A., Yang J.C. (2010). Selection of CD8+PD-1+ lymphocytes in fresh human melanomas enriches for tumor-reactive T cells. J. Immunother..

[102] Gros A., Robbins P.F., Yao X., Li Y.F., Turcotte S., Tran E., Wunderlich J.R., Mixon A., Farid S., Dudley M.E. (2014). PD-1 identifies the patient-specific CD8⁺ tumor-reactive repertoire infiltrating human tumors. J. Clin. Investig..

[103] Fernandez-Poma S.M., Salas-Benito D., Lozano T., Casares N., Riezu-Boj J.I., Mancheño U., Elizalde E., Alignani D., Zubeldia N., Otano I. (2017). Expansion of Tumor-Infiltrating CD8. Cancer Res..

[104] Yee C., Thompson J.A., Byrd D., Riddell S.R., Roche P., Celis E., Greenberg P.D. (2002). Adoptive T cell therapy using antigen-specific CD8+ T cell clones for the treatment of patients with metastatic melanoma: in vivo persistence, migration, and antitumor effect of transferred T cells. Proc. Natl. Acad. Sci. USA.

[105] Mackensen A., Meidenbauer N., Vogl S., Laumer M., Berger J., Andreesen R. (2006). Phase I study of adoptive T-cell therapy using antigen-specific CD8+ T cells for the treatment of patients with metastatic melanoma. J. Clin. Oncol..

[106] Huang J., El-Gamil M., Dudley M.E., Li Y.F., Rosenberg S.A., Robbins P.F. (2004). T cells associated with tumor regression recognize frameshifted products of the CDKN2A tumor suppressor gene locus and a mutated HLA class I gene product. J. Immunol..

[107] Lu Y.C., Yao X., Li Y.F., El-Gamil M., Dudley M.E., Yang J.C., Almeida J.R., Douek D.C., Samuels Y., Rosenberg S.A. (2013). Mutated PPP1R3B is recognized by T cells used to treat a melanoma patient who experienced a durable complete tumor regression. J. Immunol..

[108] Robbins P.F., Lu Y.C., El-Gamil M., Li Y.F., Gross C., Gartner J., Lin J.C., Teer J.K., Cliften P., Tycksen E. (2013). Mining exomic sequencing data to identify mutated antigens recognized by adoptively transferred tumor-reactive T cells. Nat. Med..

[109] Gfeller D., Bassani-Sternberg M. (2018). Predicting Antigen Presentation-What Could We Learn From a Million Peptides?. Front. Immunol..

[110] Jurtz V., Paul S., Andreatta M., Marcatili P., Peters B., Nielsen M. (2017). NetMHCpan-4.0: Improved Peptide-MHC Class I Interaction Predictions Integrating Eluted Ligand and Peptide Binding Affinity Data. J. Immunol..

[111] Richters M.M., Xia H., Campbell K.M., Gillanders W.E., Griffith O.L., Griffith M. (2019). Best practices for bioinformatic characterization of neoantigens for clinical utility. Genome Med..

[112] Lu Y.C., Yao X., Crystal J.S., Li Y.F., El-Gamil M., Gross C., Davis L., Dudley M.E., Yang J.C., Samuels Y. (2014). Efficient identification of mutated cancer antigens recognized by T cells associated with durable tumor regressions. Clin. Cancer Res..

[113] Altman J.D., Moss P.A., Goulder P.J., Barouch D.H., McHeyzer-Williams M.G., Bell J.I., McMichael A.J., Davis M.M. (1996). Phenotypic analysis of antigen-specific T lymphocytes. Science.

[114] Rodenko B., Toebes M., Hadrup S.R., van Esch W.J., Molenaar A.M., Schumacher T.N., Ovaa H. (2006). Generation of peptide-MHC class I complexes through UV-mediated ligand exchange. Nat. Protoc..

[115] Newell E.W., Klein L.O., Yu W., Davis M.M. (2009). Simultaneous detection of many T-cell specificities using combinatorial tetramer staining. Nat. Methods.

[116] Newell E.W., Sigal N., Nair N., Kidd B.A., Greenberg H.B., Davis M.M. (2013). Combinatorial tetramer staining and mass cytometry analysis facilitate T-cell epitope mapping and characterization. Nat. Biotechnol..

[117] Bentzen A.K., Marquard A.M., Lyngaa R., Saini S.K., Ramskov S., Donia M., Such L., Furness A.J., McGranahan N., Rosenthal R. (2016). Large-scale detection of antigen-specific T cells using peptide-MHC-I multimers labeled with DNA barcodes. Nat. Biotechnol..

[118] Zhang S.Q., Ma K.Y., Schonnesen A.A., Zhang M., He C., Sun E., Williams C.M., Jia W., Jiang N. (2018). High-throughput determination of the antigen specificities of T cell receptors in single cells. Nat. Biotechnol..

[119] Fehlings M., Jhunjhunwala S., Kowanetz M., O’Gorman W.E., Hegde P.S., Sumatoh H., Lee B.H., Nardin A., Becht E., Flynn S. (2019). Late-differentiated effector neoantigen-specific CD8+ T cells are enriched in peripheral blood of non-small cell lung carcinoma patients responding to atezolizumab treatment. J. Immunother. Cancer.

[120] Veatch J.R., Jesernig B.L., Kargl J., Fitzgibbon M., Lee S.M., Baik C., Martins R., Houghton A.M., Riddell S.R. (2019). Endogenous CD4 + T Cells Recognize Neoantigens in Lung Cancer Patients, Including Recurrent Oncogenic *KRAS* and *ERBB2* (*Her2*) Driver Mutations. Cancer Immunol. Res..

[121] Tran E., Turcotte S., Gros A., Robbins P.F., Lu Y.C., Dudley M.E., Wunderlich J.R., Somerville R.P., Hogan K., Hinrichs C.S. (2014). Cancer immunotherapy based on mutation-specific CD4+ T cells in a patient with epithelial cancer. Science.

[122] Tran E., Ahmadzadeh M., Lu Y.C., Gros A., Turcotte S., Robbins P.F., Gartner J.J., Zheng Z., Li Y.F., Ray S. (2015). Immunogenicity of somatic mutations in human gastrointestinal cancers. Science.

[123] Tran E., Robbins P.F., Lu Y.C., Prickett T.D., Gartner J.J., Jia L., Pasetto A., Zheng Z., Ray S., Groh E.M. (2016). T-Cell Transfer Therapy Targeting Mutant KRAS in Cancer. N. Engl. J. Med..

[124] Bobisse S., Genolet R., Roberti A., Tanyi J.L., Racle J., Stevenson B.J., Iseli C., Michel A., Le Bitoux M.A., Guillaume P. (2018). Sensitive and frequent identification of high avidity neo-epitope specific CD8. Nat. Commun..

[125] Deniger D.C., Pasetto A., Robbins P.F., Gartner J.J., Prickett T.D., Paria B.C., Malekzadeh P., Jia L., Yossef R., Langhan M.M. (2018). T-cell Responses to TP53 "Hotspot" Mutations and Unique Neoantigens Expressed by Human Ovarian Cancers. Clin. Cancer Res..

[126] Liu S., Matsuzaki J., Wei L., Tsuji T., Battaglia S., Hu Q., Cortes E., Wong L., Yan L., Long M. (2019). Efficient identification of neoantigen-specific T-cell responses in advanced human ovarian cancer. J. Immunother. Cancer.

[127] Zacharakis N., Chinnasamy H., Black M., Xu H., Lu Y.C., Zheng Z., Pasetto A., Langhan M., Shelton T., Prickett T. (2018). Immune recognition of somatic mutations leading to complete durable regression in metastatic breast cancer. Nat. Med..

[128] Meng Q., Valentini D., Rao M., Moro C.F., Paraschoudi G., Jäger E., Dodoo E., Rangelova E., Del Chiaro M., Maeurer M. (2019). Neoepitope targets of tumour-infiltrating lymphocytes from patients with pancreatic cancer. Br. J. Cancer.

[129] Scheper W., Kelderman S., Fanchi L.F., Linnemann C., Bendle G., de Rooij M.A.J., Hirt C., Mezzadra R., Slagter M., Dijkstra K. (2019). Low and variable tumor reactivity of the intratumoral TCR repertoire in human cancers. Nat. Med..

[130] Cohen C.J., Gartner J.J., Horovitz-Fried M., Shamalov K., Trebska-McGowan K., Bliskovsky V.V., Parkhurst M.R., Ankri C., Prickett T.D., Crystal J.S. (2015). Isolation of neoantigen-specific T cells from tumor and peripheral lymphocytes. J. Clin. Investig..

[131] Gros A., Parkhurst M.R., Tran E., Pasetto A., Robbins P.F., Ilyas S., Prickett T.D., Gartner J.J., Crystal J.S., Roberts I.M. (2016). Prospective identification of neoantigen-specific lymphocytes in the peripheral blood of melanoma patients. Nat. Med..

[132] Martin S.D., Wick D.A., Nielsen J.S., Little N., Holt R.A., Nelson B.H. (2017). A library-based screening method identifies neoantigen-reactive T cells in peripheral blood prior to relapse of ovarian cancer. Oncoimmunology.

[133] Gros A., Tran E., Parkhurst M.R., Ilyas S., Pasetto A., Groh E.M., Robbins P.F., Yossef R., Garcia-Garijo A., Fajardo C.A. (2019). Recognition of human gastrointestinal cancer neoantigens by circulating PD-1+ lymphocytes. J. Clin. Investig..

[134] Theaker S.M., Rius C., Greenshields-Watson A., Lloyd A., Trimby A., Fuller A., Miles J.J., Cole D.K., Peakman M., Sewell A.K. (2016). T-cell libraries allow simple parallel generation of multiple peptide-specific human T-cell clones. J. Immunol. Methods.

[135] Yossef R., Tran E., Deniger D.C., Gros A., Pasetto A., Parkhurst M.R., Gartner J.J., Prickett T.D., Cafri G., Robbins P.F. (2018). Enhanced detection of neoantigen-reactive T cells targeting unique and shared oncogenes for personalized cancer immunotherapy. JCI Insight..

[136] Cafri G., Yossef R., Pasetto A., Deniger D.C., Lu Y.C., Parkhurst M., Gartner J.J., Jia L., Ray S., Ngo L.T. (2019). Memory T cells targeting oncogenic mutations detected in peripheral blood of epithelial cancer patients. Nat. Commun..

[137] Veatch J.R., Lee S.M., Fitzgibbon M., Chow I.T., Jesernig B., Schmitt T., Kong Y.Y., Kargl J., Houghton A.M., Thompson J.A. (2018). Tumor-infiltrating BRAFV600E-specific CD4+ T cells correlated with complete clinical response in melanoma. J. Clin. Investig..

[138] Chheda Z.S., Kohanbash G., Okada K., Jahan N., Sidney J., Pecoraro M., Yang X., Carrera D.A., Downey K.M., Shrivastav S. (2018). Novel and shared neoantigen derived from histone 3 variant H3.3K27M mutation for glioma T cell therapy. J. Exp. Med..

[139] Lo W., Parkhurst M., Robbins P.F., Tran E., Lu Y.C., Jia L., Gartner J.J., Pasetto A., Deniger D., Malekzadeh P. (2019). Immunologic Recognition of a Shared p53 Mutated Neoantigen in a Patient with Metastatic Colorectal Cancer. Cancer Immunol. Res..

[140] Malekzadeh P., Pasetto A., Robbins P.F., Parkhurst M.R., Paria B.C., Jia L., Gartner J.J., Hill V., Yu Z., Restifo N.P. (2019). Neoantigen screening identifies broad TP53 mutant immunogenicity in patients with epithelial cancers. J. Clin. Investig..

[141] Morgan R.A., Dudley M.E., Wunderlich J.R., Hughes M.S., Yang J.C., Sherry R.M., Royal R.E., Topalian S.L., Kammula U.S., Restifo N.P. (2006). Cancer regression in patients after transfer of genetically engineered lymphocytes. Science.

[142] Kuball J., Dossett M.L., Wolfl M., Ho W.Y., Voss R.H., Fowler C., Greenberg P.D. (2007). Facilitating matched pairing and expression of TCR chains introduced into human T cells. Blood.

[143] Bendle G.M., Linnemann C., Hooijkaas A.I., Bies L., de Witte M.A., Jorritsma A., Kaiser A.D., Pouw N., Debets R., Kieback E. (2010). Lethal graft-versus-host disease in mouse models of T cell receptor gene therapy. Nat. Med..

[144] van Loenen M.M., de Boer R., Amir A.L., Hagedoorn R.S., Volbeda G.L., Willemze R., van Rood J.J., Falkenburg J.H., Heemskerk M.H. (2010). Mixed T cell receptor dimers harbor potentially harmful neoreactivity. Proc. Natl. Acad. Sci. USA.

[145] Leisegang M., Engels B., Meyerhuber P., Kieback E., Sommermeyer D., Xue S.A., Reuss S., Stauss H., Uckert W. (2008). Enhanced functionality of T cell receptor-redirected T cells is defined by the transgene cassette. J. Mol. Med. (Berl.).

[146] Legut M., Dolton G., Mian A.A., Ottmann O.G., Sewell A.K. (2018). CRISPR-mediated TCR replacement generates superior anticancer transgenic T cells. Blood.

[147] Roth T.L., Puig-Saus C., Yu R., Shifrut E., Carnevale J., Li P.J., Hiatt J., Saco J., Krystofinski P., Li H. (2018). Reprogramming human T cell function and specificity with non-viral genome targeting. Nature.

[148] Albers J.J., Ammon T., Gosmann D., Audehm S., Thoene S., Winter C., Secci R., Wolf A., Stelzl A., Steiger K. (2019). Gene editing enables T-cell engineering to redirect antigen specificity for potent tumor rejection. Life Sci. Alliance.

[149] Garber K. (2018). Driving T-cell immunotherapy to solid tumors. Nat. Biotechnol..

[150] Kennedy R., Celis E. (2008). Multiple roles for CD4+ T cells in anti-tumor immune responses. Immunol. Rev..

[151] Borst J., Ahrends T., Bąbała N., Melief C.J.M., Kastenmüller W. (2018). CD4 + T cell help in cancer immunology and immunotherapy. Nat. Rev. Immunol..

[152] Linnemann C., van Buuren M.M., Bies L., Verdegaal E.M., Schotte R., Calis J.J., Behjati S., Velds A., Hilkmann H., Atmioui D.E. (2015). High-throughput epitope discovery reveals frequent recognition of neo-antigens by CD4+ T cells in human melanoma. Nat. Med..

[153] Alspach E., Lussier D.M., Miceli A.P., Kizhvatov I., DuPage M., Luoma A.M., Meng W., Lichti C.F., Esaulova E., Vomund A.N. (2019). MHC-II neoantigens shape tumour immunity and response to immunotherapy. Nature.

[154] Matsuzaki J., Tsuji T., Luescher I.F., Shiku H., Mineno J., Okamoto S., Old L.J., Shrikant P., Gnjatic S., Odunsi K. (2015). Direct tumor recognition by a human CD4(+) T-cell subset potently mediates tumor growth inhibition and orchestrates anti-tumor immune responses. Sci. Rep..

[155] Abelin J.G., Harjanto D., Malloy M., Suri P., Colson T., Goulding S.P., Creech A.L., Serrano L.R., Nasir G., Nasrullah Y. (2019). Defining HLA-II Ligand Processing and Binding Rules with Mass Spectrometry Enhances Cancer Epitope Prediction. Immunity.

[156] Moeller M., Haynes N.M., Kershaw M.H., Jackson J.T., Teng M.W., Street S.E., Cerutti L., Jane S.M., Trapani J.A., Smyth M.J. (2005). Adoptive transfer of gene-engineered CD4+ helper T cells induces potent primary and secondary tumor rejection. Blood.

[157] Wang L.X., Shu S., Disis M.L., Plautz G.E. (2007). Adoptive transfer of tumor-primed, in vitro-activated, CD4+ T effector cells (TEs) combined with CD8+ TEs provides intratumoral TE proliferation and synergistic antitumor response. Blood.

[158] Hunder N.N., Wallen H., Cao J., Hendricks D.W., Reilly J.Z., Rodmyre R., Jungbluth A., Gnjatic S., Thompson J.A., Yee C. (2008). Treatment of metastatic melanoma with autologous CD4+ T cells against NY-ESO-1. N. Engl. J. Med..

[159] Crowther M.D., Dolton G., Legut M., Caillaud M.E., Lloyd A., Attaf M., Galloway S.A.E., Rius C., Farrell C.P., Szomolay B. (2020). Genome-wide CRISPR-Cas9 screening reveals ubiquitous T cell cancer targeting via the monomorphic MHC class I-related protein MR1. Nat. Immunol..

[160] Gold M.C., Cerri S., Smyk-Pearson S., Cansler M.E., Vogt T.M., Delepine J., Winata E., Swarbrick G.M., Chua W.J., Yu Y.Y. (2010). Human mucosal associated invariant T cells detect bacterially infected cells. PLoS Biol..

[161] Le Bourhis L., Dusseaux M., Bohineust A., Bessoles S., Martin E., Premel V., Coré M., Sleurs D., Serriari N.E., Treiner E. (2013). MAIT cells detect and efficiently lyse bacterially-infected epithelial cells. PLoS Pathog..

[162] Lepore M., Kalinichenko A., Calogero S., Kumar P., Paleja B., Schmaler M., Narang V., Zolezzi F., Poidinger M., Mori L. (2017). Functionally diverse human T cells recognize non-microbial antigens presented by MR1. Elife.

[163] Kaech S.M., Cui W. (2012). Transcriptional control of effector and memory CD8+ T cell differentiation. Nat. Rev. Immunol..

[164] Klebanoff C.A., Gattinoni L., Restifo N.P. (2012). Sorting through subsets: which T-cell populations mediate highly effective adoptive immunotherapy?. J. Immunother..

[165] Marek N., Myśliwiec M., Raczyńska K., Zorena K., Myśliwska J., Trzonkowski P. (2010). Increased spontaneous production of VEGF by CD4+ T cells in type 1 diabetes. Clin. Immunol..

[166] Berger C., Jensen M.C., Lansdorp P.M., Gough M., Elliott C., Riddell S.R. (2008). Adoptive transfer of effector CD8+ T cells derived from central memory cells establishes persistent T cell memory in primates. J. Clin. Investig..

[167] Klebanoff C.A., Gattinoni L., Torabi-Parizi P., Kerstann K., Cardones A.R., Finkelstein S.E., Palmer D.C., Antony P.A., Hwang S.T., Rosenberg S.A. (2005). Central memory self/tumor-reactive CD8+ T cells confer superior antitumor immunity compared with effector memory T cells. Proc. Natl. Acad. Sci. USA.

[168] Zhou J., Shen X., Huang J., Hodes R.J., Rosenberg S.A., Robbins P.F. (2005). Telomere length of transferred lymphocytes correlates with in vivo persistence and tumor regression in melanoma patients receiving cell transfer therapy. J. Immunol..

[169] Hinrichs C.S., Borman Z.A., Cassard L., Gattinoni L., Spolski R., Yu Z., Sanchez-Perez L., Muranski P., Kern S.J., Logun C. (2009). Adoptively transferred effector cells derived from naive rather than central memory CD8+ T cells mediate superior antitumor immunity. Proc. Natl. Acad. Sci. USA.

[170] Wu F., Zhang W., Shao H., Bo H., Shen H., Li J., Liu Y., Wang T., Ma W., Huang S. (2013). Human effector T cells derived from central memory cells rather than CD8(+)T cells modified by tumor-specific TCR gene transfer possess superior traits for adoptive immunotherapy. Cancer Lett..

[171] Klebanoff C.A., Scott C.D., Leonardi A.J., Yamamoto T.N., Cruz A.C., Ouyang C., Ramaswamy M., Roychoudhuri R., Ji Y., Eil R.L. (2016). Memory T cell-driven differentiation of naive cells impairs adoptive immunotherapy. J. Clin. Investig..

[172] Gattinoni L., Klebanoff C.A., Restifo N.P. (2012). Paths to stemness: building the ultimate antitumour T cell. Nat. Rev. Cancer.

[173] Gattinoni L., Lugli E., Ji Y., Pos Z., Paulos C.M., Quigley M.F., Almeida J.R., Gostick E., Yu Z., Carpenito C. (2011). A human memory T cell subset with stem cell-like properties. Nat. Med..

[174] Gattinoni L., Speiser D.E., Lichterfeld M., Bonini C. (2017). T memory stem cells in health and disease. Nat. Med..

[175] Cieri N., Camisa B., Cocchiarella F., Forcato M., Oliveira G., Provasi E., Bondanza A., Bordignon C., Peccatori J., Ciceri F. (2013). IL-7 and IL-15 instruct the generation of human memory stem T cells from naive precursors. Blood.

[176] Levine B.L., Bernstein W.B., Connors M., Craighead N., Lindsten T., Thompson C.B., June C.H. (1997). Effects of CD28 costimulation on long-term proliferation of CD4+ T cells in the absence of exogenous feeder cells. J. Immunol..

[177] Maus M.V., Thomas A.K., Leonard D.G., Allman D., Addya K., Schlienger K., Riley J.L., June C.H. (2002). Ex vivo expansion of polyclonal and antigen-specific cytotoxic T lymphocytes by artificial APCs expressing ligands for the T-cell receptor, CD28 and 4-1BB. Nat. Biotechnol..

[178] Butler M.O., Hirano N. (2014). Human cell-based artificial antigen-presenting cells for cancer immunotherapy. Immunol. Rev..

[179] Suhoski M.M., Golovina T.N., Aqui N.A., Tai V.C., Varela-Rohena A., Milone M.C., Carroll R.G., Riley J.L., June C.H. (2007). Engineering artificial antigen-presenting cells to express a diverse array of co-stimulatory molecules. Mol. Ther..

[180] Marek-Trzonkowska N., Myśliwiec M., Iwaszkiewicz-Grześ D., Gliwiński M., Derkowska I., Żalińska M., Zieliński M., Grabowska M., Zielińska H., Piekarska K. (2016). Factors affecting long-term efficacy of T regulatory cell-based therapy in type 1 diabetes. J. Transl. Med..

[181] Li Y., Kurlander R.J. (2010). Comparison of anti-CD3 and anti-CD28-coated beads with soluble anti-CD3 for expanding human T cells: differing impact on CD8 T cell phenotype and responsiveness to restimulation. J. Transl. Med..

[182] Kagoya Y., Nakatsugawa M., Ochi T., Cen Y., Guo T., Anczurowski M., Saso K., Butler M.O., Hirano N. (2017). Transient stimulation expands superior antitumor T cells for adoptive therapy. JCI Insight..

[183] Gattinoni L., Powell D.J., Rosenberg S.A., Restifo N.P. (2006). Adoptive immunotherapy for cancer: building on success. Nat. Rev. Immunol..

[184] Petrozziello E., Sturmheit T., Mondino A. (2015). Exploiting cytokines in adoptive T-cell therapy of cancer. Immunotherapy.

[185] Dwyer C.J., Knochelmann H.M., Smith A.S., Wyatt M.M., Rangel Rivera G.O., Arhontoulis D.C., Bartee E., Li Z., Rubinstein M.P., Paulos C.M. (2019). Fueling Cancer Immunotherapy With Common Gamma Chain Cytokines. Front. Immunol..

[186] Gliwiński M., Piotrowska M., Iwaszkiewicz-Grześ D., Urban-Wójciuk Z., Trzonkowski P. (2019). Therapy with CD4+CD25+ T regulatory cells – should we be afraid of cancer?. Contemp. Oncol. (Pozn.).

[187] Liao W., Lin J.X., Leonard W.J. (2013). Interleukin-2 at the crossroads of effector responses, tolerance, and immunotherapy. Immunity.

[188] Nguyen L.T., Saibil S.D., Sotov V., Le M.X., Khoja L., Ghazarian D., Bonilla L., Majeed H., Hogg D., Joshua A.M. (2019). Phase II clinical trial of adoptive cell therapy for patients with metastatic melanoma with autologous tumor-infiltrating lymphocytes and low-dose interleukin-2. Cancer Immunol. Immunother..

[189] Rosenzwajg M., Lorenzon R., Cacoub P., Pham H.P., Pitoiset F., El Soufi K., RIbet C., Bernard C., Aractingi S., Banneville B. (2019). Immunological and clinical effects of low-dose interleukin-2 across 11 autoimmune diseases in a single, open clinical trial. Ann. Rheum. Dis..

[190] Saadoun D., Rosenzwajg M., Joly F., Six A., Carrat F., Thibault V., Sene D., Cacoub P., Klatzmann D. (2011). Regulatory T-cell responses to low-dose interleukin-2 in HCV-induced vasculitis. N. Engl. J. Med..

[191] Klatzmann D., Abbas A.K. (2015). The promise of low-dose interleukin-2 therapy for autoimmune and inflammatory diseases. Nat. Rev. Immunol..

[192] Heemskerk B., Liu K., Dudley M.E., Johnson L.A., Kaiser A., Downey S., Zheng Z., Shelton T.E., Matsuda K., Robbins P.F. (2008). Adoptive cell therapy for patients with melanoma, using tumor-infiltrating lymphocytes genetically engineered to secrete interleukin-2. Hum. Gene. Ther..

[193] Sockolosky J.T., Trotta E., Parisi G., Picton L., Su L.L., Le A.C., Chhabra A., Silveria S.L., George B.M., King I.C. (2018). Selective targeting of engineered T cells using orthogonal IL-2 cytokine-receptor complexes. Science.

[194] Sun Z., Ren Z., Yang K., Liu Z., Cao S., Deng S., Xu L., Liang Y., Guo J., Bian Y. (2019). A next-generation tumor-targeting IL-2 preferentially promotes tumor-infiltrating CD8. Nat. Commun..

[195] Caserta S., Alessi P., Basso V., Mondino A. (2010). IL-7 is superior to IL-2 for ex vivo expansion of tumour-specific CD4(+) T cells. Eur. J. Immunol..

[196] Rosenberg S.A., Sportès C., Ahmadzadeh M., Fry T.J., Ngo L.T., Schwarz S.L., Stetler-Stevenson M., Morton K.E., Mavroukakis S.A., Morre M. (2006). IL-7 administration to humans leads to expansion of CD8+ and CD4+ cells but a relative decrease of CD4+ T-regulatory cells. J. Immunother..

[197] Sportès C., Hakim F.T., Memon S.A., Zhang H., Chua K.S., Brown M.R., Fleisher T.A., Krumlauf M.C., Babb R.R., Chow C.K. (2008). Administration of rhIL-7 in humans increases in vivo TCR repertoire diversity by preferential expansion of naive T cell subsets. J. Exp. Med..

[198] Ding Z.C., Habtetsion T., Cao Y., Li T., Liu C., Kuczma M., Chen T., Hao Z., Bryan L., Munn D.H. (2017). Adjuvant IL-7 potentiates adoptive T cell therapy by amplifying and sustaining polyfunctional antitumor CD4+ T cells. Sci. Rep..

[199] Robinson T.O., Schluns K.S. (2017). The potential and promise of IL-15 in immuno-oncogenic therapies. Immunol. Lett..

[200] Tang L., Zheng Y., Melo M.B., Mabardi L., Castaño A.P., Xie Y.Q., Li N., Kudchodkar S.B., Wong H.C., Jeng E.K. (2018). Enhancing T cell therapy through TCR-signaling-responsive nanoparticle drug delivery. Nat. Biotechnol..

[201] Kunert A., Chmielewski M., Wijers R., Berrevoets C., Abken H., Debets R. (2017). Intra-tumoral production of IL18, but not IL12, by TCR-engineered T cells is non-toxic and counteracts immune evasion of solid tumors. Oncoimmunology.

[202] Chen Y., Yu F., Jiang Y., Chen J., Wu K., Chen X., Lin Y., Zhang H., Li L., Zhang Y. (2018). Adoptive Transfer of Interleukin-21-stimulated Human CD8+ T Memory Stem Cells Efficiently Inhibits Tumor Growth. J. Immunother..

[203] Santegoets S.J., Turksma A.W., Suhoski M.M., Stam A.G., Albelda S.M., Hooijberg E., Scheper R.J., van den Eertwegh A.J., Gerritsen W.R., Powell D.J. (2013). IL-21 promotes the expansion of CD27+ CD28+ tumor infiltrating lymphocytes with high cytotoxic potential and low collateral expansion of regulatory T cells. J. Transl. Med..

[204] Zhang H., Snyder K.M., Suhoski M.M., Maus M.V., Kapoor V., June C.H., Mackall C.L. (2007). 4-1BB is superior to CD28 costimulation for generating CD8+ cytotoxic lymphocytes for adoptive immunotherapy. J. Immunol..

[205] Chacon J.A., Wu R.C., Sukhumalchandra P., Molldrem J.J., Sarnaik A., Pilon-Thomas S., Weber J., Hwu P., Radvanyi L. (2013). Co-stimulation through 4-1BB/CD137 improves the expansion and function of CD8(+) melanoma tumor-infiltrating lymphocytes for adoptive T-cell therapy. PLoS One.

[206] Weigelin B., Bolaños E., Teijeira A., Martinez-Forero I., Labiano S., Azpilikueta A., Morales-Kastresana A., Quetglas J.I., Wagena E., Sánchez-Paulete A.R. (2015). Focusing and sustaining the antitumor CTL effector killer response by agonist anti-CD137 mAb. Proc. Natl. Acad. Sci. USA.

[207] Chester C., Sanmamed M.F., Wang J., Melero I. (2018). Immunotherapy targeting 4-1BB: mechanistic rationale, clinical results, and future strategies. Blood.

[208] Song A., Song J., Tang X., Croft M. (2007). Cooperation between CD4 and CD8 T cells for anti-tumor activity is enhanced by OX40 signals. Eur. J. Immunol..

[209] Wu X., Gu Z., Chen Y., Chen B., Chen W., Weng L., Liu X. (2019). Application of PD-1 Blockade in Cancer Immunotherapy. Comput. Struct. Biotechnol. J..

[210] Wei S.C., Duffy C.R., Allison J.P. (2018). Fundamental Mechanisms of Immune Checkpoint Blockade Therapy. Cancer Discov..

[211] Peng W., Liu C., Xu C., Lou Y., Chen J., Yang Y., Yagita H., Overwijk W.W., Lizée G., Radvanyi L. (2012). PD-1 blockade enhances T-cell migration to tumors by elevating IFN-γ inducible chemokines. Cancer Res..

[212] Chapuis A.G., Roberts I.M., Thompson J.A., Margolin K.A., Bhatia S., Lee S.M., Sloan H.L., Lai I.P., Farrar E.A., Wagener F. (2016). T-Cell Therapy Using Interleukin-21-Primed Cytotoxic T-Cell Lymphocytes Combined With Cytotoxic T-Cell Lymphocyte Antigen-4 Blockade Results in Long-Term Cell Persistence and Durable Tumor Regression. J. Clin. Oncol..

[213] Shi L.Z., Goswami S., Fu T., Guan B., Chen J., Xiong L., Zhang J., Ng Tang D., Zhang X., Vence L. (2019). Blockade of CTLA-4 and PD-1 Enhances Adoptive T-cell Therapy Efficacy in an ICOS-Mediated Manner. Cancer Immunol. Res..

[214] Mardiana S., Solomon B.J., Darcy P.K., Beavis P.A. (2019). Supercharging adoptive T cell therapy to overcome solid tumor-induced immunosuppression. Sci. Transl. Med..

[215] Poschke I., Lövgren T., Adamson L., Nyström M., Andersson E., Hansson J., Tell R., Masucci G.V., Kiessling R. (2014). A phase I clinical trial combining dendritic cell vaccination with adoptive T cell transfer in patients with stage IV melanoma. Cancer Immunol. Immunother..

[216] Chodon T., Comin-Anduix B., Chmielowski B., Koya R.C., Wu Z., Auerbach M., Ng C., Avramis E., Seja E., Villanueva A. (2014). Adoptive transfer of MART-1 T-cell receptor transgenic lymphocytes and dendritic cell vaccination in patients with metastatic melanoma. Clin. Cancer Res..

[217] Nowicki T.S., Berent-Maoz B., Cheung-Lau G., Huang R.R., Wang X., Tsoi J., Kaplan-Lefko P., Cabrera P., Tran J., Pang J. (2019). A Pilot Trial of the Combination of Transgenic NY-ESO-1-reactive Adoptive Cellular Therapy with Dendritic Cell Vaccination with or without Ipilimumab. Clin. Cancer Res..

[218] Idorn M., Skadborg S.K., Kellermann L., Halldórsdóttir H.R., Holmen Olofsson G., Met Ö., Thor Straten P. (2018). Chemokine receptor engineering of T cells with CXCR2 improves homing towards subcutaneous human melanomas in xenograft mouse model. Oncoimmunology.

[219] Peng W., Ye Y., Rabinovich B.A., Liu C., Lou Y., Zhang M., Whittington M., Yang Y., Overwijk W.W., Lizée G. (2010). Transduction of tumor-specific T cells with CXCR2 chemokine receptor improves migration to tumor and antitumor immune responses. Clin. Cancer Res..

[220] Ho P.C., Bihuniak J.D., Macintyre A.N., Staron M., Liu X., Amezquita R., Tsui Y.C., Cui G., Micevic G., Perales J.C. (2015). Phosphoenolpyruvate Is a Metabolic Checkpoint of Anti-tumor T Cell Responses. Cell.

[221] Scharping N.E., Menk A.V., Moreci R.S., Whetstone R.D., Dadey R.E., Watkins S.C., Ferris R.L., Delgoffe G.M. (2016). The Tumor Microenvironment Represses T Cell Mitochondrial Biogenesis to Drive Intratumoral T Cell Metabolic Insufficiency and Dysfunction. Immunity.

[222] Kishton R.J., Sukumar M., Restifo N.P. (2017). Metabolic Regulation of T Cell Longevity and Function in Tumor Immunotherapy. Cell Metab..

[223] Kouidhi S., Ben Ayed F., Benammar Elgaaied A. (2018). Targeting Tumor Metabolism: A New Challenge to Improve Immunotherapy. Front. Immunol..

[224] Addou-Klouche L., Aribi M. (2017). NK Cells in Cancer Immunotherapy. Natural Killer Cells.

[225] Shimasaki N., Jain A., Campana D. (2020). NK cells for cancer immunotherapy. Nat. Rev. Drug Discov..

